# Aminoguanidine-based bioactive proligand as AIEE probe for anticancer and anticovid studies†

**DOI:** 10.1039/d4ra00554f

**Published:** 2024-04-25

**Authors:** K. K. Mohammed Hashim, E. Manoj

**Affiliations:** a Department of Applied Chemistry, Cochin University of Science and Technology Kochi Kerala 682 022 India manoje@cusat.ac.in

## Abstract

The emission features of a novel bioactive compound, 1,3-bis(2-hydroxy-3,5-diiodophenyl-methylideneamino)guanidine is found impressive with aggregation induced emission enhancement. The nitrogen and iodine rich multidentate proligand was characterized physicochemically. SCXRD and Hirshfeld surface investigation have revealed the presence of significant triangular iodine bonding apart from hydrogen bonding, weak C–H⋯π and π⋯π intermolecular interactions. These interactions collectively contribute to the solid-state packing arrangement of the molecules within the crystal lattice. The band gap of the compound was estimated experimentally and is supported with theoretical calculations. The solid-state fluorescence quantum yield of *Φ* = 0.36 emphasizes the utility of the proligand and the AIEE characteristics is attributed to restricted intramolecular motions as indicated by fluorescence lifetime decay studies. Strong interaction of the compound with calf thymus DNA was explored experimentally and found to align with *in silico* docking results. Notably, *in vitro* anticancer assessment on MCF-7 breast cancer cells show an IC_50_ value of 181.05 μg mL^−1^ and signifying its potent cytotoxic properties. Also, the compound is found to have lesser cytotoxicity against L929 normal cell line with an IC_50_ value of 356.54 μg mL^−1^. Computational studies further underscore the exceptional binding affinity with active sites in the SARS-CoV-2 main protease 3CL^pro^, surpassing established repurposed drugs. Furthermore, the proligand demonstrates excellent putative affinity towards the SARS-CoV-2 spike glycoprotein, accompanied by its distinctive AIEE attributes, drug likeness and DNA binding capability rendering it a valuable tool for prospective research investigations.

## Abstract 2

An anticancer proligand, with AIEE features, demonstrates exceptional binding efficiencies with DNA and SARS-CoV-2 and offers as a promising bioprobe.

## Graphical abstract

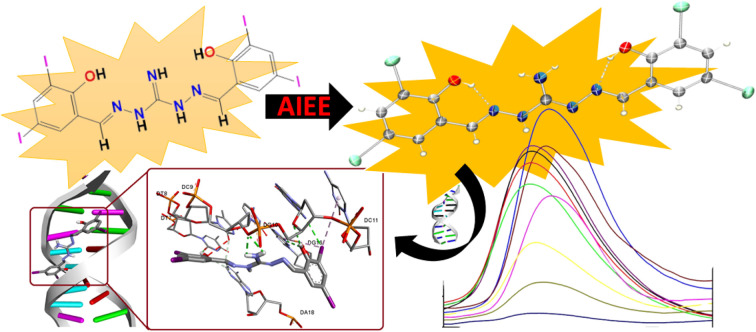

## Introduction

The emergence of the aggregation induced emission (AIE) phenomenon has attracted much research interest in the scientific community due to its wide range of applications such as chemosensors, biomedical imaging, photodynamic therapy, and optoelectronic devices.^1–7^ Typically, the AIE properties of organic compounds arise from factors like restriction of intramolecular rotations (RIR), restricted intramolecular vibrations (RIV), the formation of J- or H-aggregates, intramolecular planarization, *cis*–*trans* isomerization, twisted intramolecular charge transfer (TICT) *etc.*^8–10^ The synthesis of new luminescent materials has been of significant interest, and a lot of fluorescent bioprobes have been designed and developed for fluorescence imaging and made remarkable impacts on the biological reseachers.^11^ Fluorescent probes facilitate the visualization of dynamic biological practices in living cells and organisms, providing valuable insights into intricate cellular activities through their ability to emit fluorescence signals.^12^ There is a growing demand for fluorescent probes that can enable long-term tracking of specific enzymes or biomarkers, facilitating early detection of diseases. Fluorescent probes utilizing organic compounds have the potential to be valuable for monitoring biological processes in both *in vitro* and *in vivo*.^13^ Luminescent nanoparticles are also currently garnering significant interest in photoluminescence (PL) imaging due to their improved physicochemical stability and adaptable surface and optical characteristics, making them versatile tools for multifunctional bioimaging.^14,15^ Fluorescence probes designed for bio-imaging within the near-infrared spectrum (650–900 nm [NIR-I] and 1000–1700 nm [NIR-II]) have gained significant attention and are customized for analyzing biological components or microenvironmental factors.^12,16^ These NIR fluorophores, in contrast to visible emissions, significantly diminish background interference and attain greater tissue penetration, thereby improving imaging capabilities.^17^ NIR fluorescent probes are anticipated to achieve sensitive and real-time detection, facilitating precise assessment of biological activity with high specificity. Though NIR-II spectrum fluorescence imaging holds promise for exploring deep-tissue biology, the main challenge in bio-imaging lies in the low availability of NIR-II fluorophores that combine high brightness and biocompatibility effectively.^12^ Currently, the development of organic fluorophores tailored for NIR-II imaging, particularly for monitoring *in vivo* activities, presents a significant challenge.^18^ Its applications range from improved bioimaging to the development of innovative biosensors and the deeper exploration of biomolecular interactions.^5,8^ AIE luminogen's distinctive ability to enhance fluorescence upon aggregation offers a range of exciting possibilities for anticancer applications.^19–21^ Also, the investigation of new luminescent probes capable of binding to SARS-CoV-2 holds immense potential for enhancing the sensitivity of coronavirus detection. The identification of SARS-CoV-2 primarily revolves around viral nucleic acids, antigens, and immune responses. Utilizing techniques like polymerase chain reaction (PCR) or DNA-RNA hybridization, the nucleic acid detection method identifies viral RNA.^22,23^ Despite being the preferred method for early diagnosis, nucleic acid detection is both expensive and time-consuming.^23^ Recent studies have shown widespread adoption of AIE-based fluorescent bioprobes in biomedical fields, particularly for their simplicity, rapidity, and accuracy in early detection of SARS-CoV-2.^24–28^ AIE luminescent probes offer high sensitivity, specificity, and stability, facilitating efficient viral detection in various biological samples, aiding in timely disease management. Compounds that demonstrate the AIE phenomenon possess significant potential as valuable assets in the battle against COVID-19.^8,28^

Many fluorogenic molecules face synthetic complexities. Our aim is to design bioactive compound that exhibit AIE effects using simple synthetic method. Considering this, Schiff base AIEE compounds are intriguing due to their simple synthesis and remarkable fluorescent properties, offering potential applications in diverse fields like biological, sensors and optoelectronics.^10,29,30^ Aminoguanidine-based Schiff-base compounds are biologically significant due to their versatile properties, holding promise in disease treatment, drug development, and various aspects of biological research.^31,32^ Herein, we report a novel 1,3-diaminoguanidine based luminogen, and is characterized by single crystal X-ray diffraction (SCXRD), MALDI-TOF MS, ^1^H-NMR, ^13^C-NMR, IR and UV-visible spectroscopic techniques. It is widely recognized that the compound's DNA binding affinity may initiate cytotoxicity in cancer cells. Therefore, a thorough examination of the impact of the synthesized compound on cancerous cells becomes imperative. In this context, the breast cancer cell line MCF-7 was utilized to systematically investigate its anticancer activities. Molecular docking studies were performed with B-DNA in order to support the experimental DNA binding results. Furthermore, as the new compound displaying fluorescent AIEE characteristics and possess similar structural features of bioactive bis(thio)carbohydrazones with exceptional putative binding efficacies,^8^ its potential utility as a novel bioprobe for studying interactions with the SARS-CoV-2 spike glycoprotein is also explored.

## Experimental

### Materials

3,5-Diiodosalicylaldehyde (Aldrich), 1,3-diaminoguanidine (Aldrich), CT-DNA, Tris base, methanol (Merck), DMSO (Spectrochem), glacial acetic acid (Spectrochem), *etc.* were used as received.

### Synthesis of 1,3-bis(2-hydroxy-3,5-diiodophenyl-methylideneamino)guanidine (H_5_L)

A hot solution of 3,5-diiodosalicylaldehyde (2.6 mmol; 973 mg) in 10 mL of methanol was added to a hot solution of 1,3-diaminoguanidine (1.25 mmol; 157 mg) in 10 mL methanol. A drop of glacial acetic acid is added and refluxed for 2 hours. White precipitate formed was kept 2 days for complete precipitation. Filtered and washed with methanol. Single crystals suitable for X-ray analysis were obtained by slow evaporation of a DMSO solution of H_5_L. Yield: 94%. CHN (calculated for H_5_L·2H_2_O): observed (calc.): C, 21.03 (21.53); H, 1.31 (1.81); N, 8.03 (8.37) %. ^1^H NMR (600 MHz, DMSO-d_6_) *δ*, ppm: 12.34 (2H, s, N–NH–C), 10.21 (2H, s, OH), 8.67 (2H, HC=N), 8.56 (1H, s, C=NH), 8.26 (2H, s, Ar–H), 8.25 (2H, s, Ar–H) (Fig. S1†). ^13^C NMR (150 MHz, DMSO-d_6_) *δ*, ppm: 155.77 (C–O), 152.95 (C=NH), 146.66 (C=N)_azomethine_, 148.04, 137.00, 123.66, 91.26, 84.77 (aromatic carbons) (Fig. S2†). MALDI-MS *m*/*z* [found (calc.)]: 802.011 (801.716) {[H_5_L + H]^+^} (Fig. S3†).

### Methods and instrumentation

Elementar Vario EL III CHNS analyzer was used for the CHN analysis of the compound. MALDI mass spectrum was taken using Bruker Autoflex spectrometer at Sophisticated Test and Instrumentation Centre (STIC), CUSAT, Kochi, India. Electronic spectrum (200–900 nm) was recorded on a UV-Thermo scientific evolution 220 spectrometer and the diffuse reflectance UV-visible spectral (UV-DRS) data were recorded on Ocean Optics DH-2000-BAL instrument at the Department of Applied Chemistry (DAC), CUSAT. The NMR spectra of the proligand H_5_L was recorded on a Bruker Avance-III HD spectrometer at 600 MHz for ^1^H NMR and 150 MHz for ^13^C NMR at NCBS-TIFR, Bangalore, India. Infrared spectrum in the range between 4000 and 400 cm^−1^ was recorded on a JASCO FT-IR 4100 spectrometer with KBr pellets and the fluorescence emission studies were conducted on a Horiba fluorolog 3 (FL-1057) Spectrofluorimeter and Jazz Ocean Optics Spectrofluorimeter at the DAC.

### X-ray crystallography

The SCXRD was carried out using Bruker SMART APEXII CCD diffractometer, equipped with a graphite crystal incident-beam monochromator, and a fine focus sealed tube with Mo Kα (*λ* = 0.71073 Å) radiation as the X-ray source at the SAIF, IIT Madras. The unit cell dimensions were measured, and the data collection was performed at 297(2) K. The Bruker SMART software and Bruker SAINT software were used for data acquisition and data integration respectively.^33^ The structure was solved by direct methods and refined by full-matrix least-squares refinement on *F*^2^ using SHELXL-2018/1 software package.^34^ The molecular and crystal structure was plotted using ORTEP,^35^ PLATON,^36^ and Mercury^37^ programs. Anisotropic refinements were performed for all non-hydrogen atoms, and all hydrogen atoms on carbon were placed in calculated positions, guided by different maps and refined isotropically. The crystal data and structural refinement parameters of the compound are given in Table 1. Experimental and theoretical bond lengths and bond angles are listed in Table S1.†

**Table 1:** Crystal data and structural refinement parameters of the compound

Parameters	H_5_L
CCDC number	2151232
Empirical formula	C_17_H_17_I_4_N_5_O_3_S
Formula weight (M)	879.01
Temperature (T)	297(2) K
Wavelength (Mo Kα)	071073 Å
Crystal system	Monoclinic
Space group	*P*2_1_/*m*
Unit cell dimensions	*a* = 9.4024(3) Å, *α* = 90°; *b* = 6.9264(2) Å, *β* = 100.0970(10)°; *c* = 19.9477(6) Å, *γ* = 90°
Volume V, Z	1278.97(7) Å^3^, 2
Calculated density (*ρ*)	2.283 mg m^−3^
Absorption coefficient, *μ*	4.981 mm^−1^
*F*(000)	812
Crystal size	0.200 × 0.150 × 0.100 mm^3^
Limiting indices	−14 ≤ *h* ≤ 14, −10 ≤ *k* ≤ 10, −30 ≤ *l* ≤ 30
Reflections collected	60 045
Independent reflections	5213 [*R*(int) = 0.0455]
Refinement method	Full-matrix least-squares on *F*_2_
Data/restraints/parameters	5213/0/181
Goodness-of-fit on *F*^2^	1.107
Final *R* indices [*I* > 2*σ* (*I*)]	*R*_1_ = 0.0394, w*R*_2_ = 0.0819
*R* indices (all data)	*R*_1_ = 0.0569, w*R*_2_ = 0.0888
Largest difference peak and hole	1.742 and −1.608 e Å^−3^

### Computational study

The density functional theory (DFT) calculations were performed using Gaussian 09 program package^38^ and GaussView 5.09 molecular visualization programs^39^ at the computational chemistry facility lab, DAC, CUSAT. Geometry optimizations and frequency calculations for the compound were performed using the B3LYP hybrid functional, incorporating Becke's three-parameter nonlocal exchange function^40^ and the Lee–Yang–Parr correlation function^41^ using the LanL2DZ basis set for the iodine atom and 6-311G(d,p) for other atoms.

### Fluorescence quantum yield study

The fluorescence quantum yield of the proligand H_5_L in solution phase was determined by referencing it to quinine sulfate (*Φ* = 0.54) in 0.1 N H_2_SO_4_, employing the given equation.^42,43^1
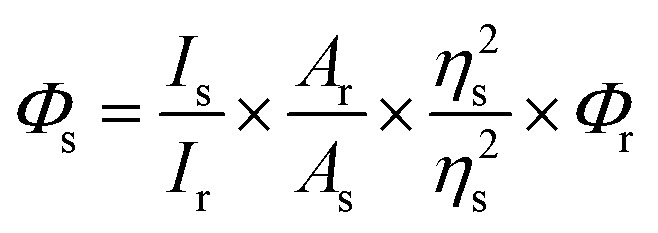


*Φ*_s_ and *Φ*_r_ represent the quantum yield of the sample and reference, respectively. *I*_s_ is the emission integrated peak area of the sample, *I*_r_ is the emission integrated peak area of the reference. *A*_r_ is the absorption maxima of the reference, *A*_s_ is the absorption maxima of the sample. *η*_s_ and *η*_r_ represent the refractive index of the sample medium and reference medium, respectively.

The radiative (*K*_r_) and non-radiative (*K*_nr_) decay constants were calculated from the quantum yield and average life time (*τ*) values using the following equations.^44^

2 *K*_r_ = (*Φ*/*τ*)

3
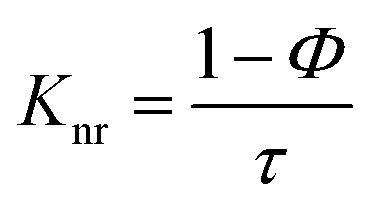


### DNA binding studies

#### Absorption spectral study

Electronic absorption spectroscopy is a versatile method employed to explore how molecules bind to DNA.^45^ The interaction between the compound and calf thymus DNA (CT-DNA) was examined in a tris-HCl buffer (pH = 7.4) at room temperature, and the concentration of CT-DNA was determined at 260 nm. A UV absorbance ratio of around 1.8–1.9 at 260 and 280 nm indicated the absence of protein contamination in the DNA.^46^ The DNA stock solution was stored at 4 °C and utilized within a span of four days after preparation. The electronic absorption titration experiment was conducted by maintaining a constant compound concentration (50 μM DMF solution) and a gradual increment of CT-DNA buffer solution (12–70 μM). The absorption spectral titration data were utilized to calculate the intrinsic binding constant (*K*_b_) for the interaction between the compound and DNA, using eqn (4).^47^4
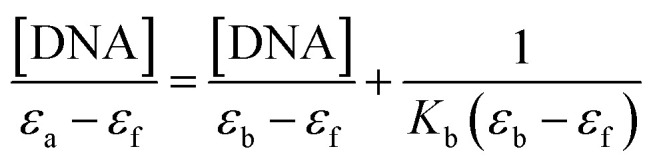
where *ε*_a_, *ε*_b_, and *ε*_f_ represents the extinction coefficient observed at a given DNA concentration, the extinction coefficient of the compound when it is fully bound to DNA, and the extinction coefficient of the free compound respectively. The plot of [DNA]/(*ε*_a_ − *ε*_f_) against [DNA] gave a straight line with the slope of 1/(*ε*_b_ − *ε*_f_), and an intercept corresponds to 1/(*K*_b_ (*ε*_b_ − *ε*_f_)). The *K*_b_ can be calculated from the slope to intercept ratio.

#### Fluorescence study

The fluorescence titration experiment was conducted to elucidate the binding nature between the compound and CT-DNA. The DNA was pre-treated with the standard intercalator ethidium bromide (EB), inducing elevated fluorescence. Subsequent addition of the compound led to the progressive quenching of fluorescence. This quenching phenomenon is explained through the Stern–Volmer equation.^48^5
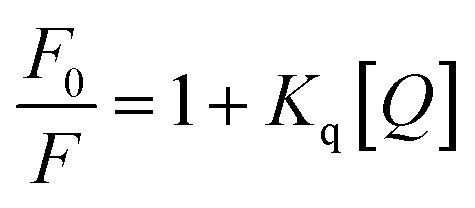
where *F* and *F*_0_ are the fluorescence intensity in the presence and absence of the compound, respectively. [*Q*] denotes the concentration of the compound and *K*_q_ signifies the linear Stern–Volmer quenching constant derived from the slope of the plot of *F*_0_/*F vs.* [*Q*].

#### Viscosity study

The viscosity experiments were carried out using an Ostwald viscometer at a constant temperature of 25.0 °C. The CT-DNA concentration in buffer was fixed at 50 μM, with the incremental addition of compound (0–140 μM). Flow times for the solutions were recorded using a digital stopwatch and replicated twice. Plotting relative viscosity (*η*/*η*_0_)^1/3^*versus* the ratio [compound]/[DNA] yielded a curve, where *η*_0_ and *η* denote the specific viscosity of free CT-DNA and the CT-DNA-compound adduct, respectively. Specific viscosity *η*_0_ and *η* where derived from the formula [(*t* − *t*_0_)/*t*_0_]. Where *t* represent the observed flow time and *t*_0_ is the buffer flow time.^49^

#### *In vitro* cytotoxicity

The MCF-7 (human breast cancer) and L929 (mouse fibroplast) cell lines were initially acquired from the National Centre for Cell Sciences (NCCS), Pune, India. The cells were cultured in Dulbecco's Modified Eagle's Medium (DMEM) obtained from Sigma-Aldrich, USA, and maintained in 25 cm^2^ tissue culture flasks. The culture medium was supplemented with 10% Fetal Bovine Serum (FBS), l-glutamine, sodium bicarbonate and an antibiotic solution comprising penicillin (100 μg mL^−1^), streptomycin (100 μg mL^−1^) and amphoteracin B (2.5 μg mL^−1^). The cell cultures were maintained at 37 °C in a humidified 5% CO_2_ incubator provided by NBS Eppendorf, Germany. Cell viability was assessed through direct observation under an inverted phase-contrast microscope and subsequently evaluated using the MTT assay method. The compound was first dissolved in 1 mL of 0.1% DMSO, and its cytotoxicity against MCF-7 and L929 cell lines were evaluated in DMEM.

#### Molecular docking study

The molecular docking simulations were performed using AutoDock Tool (ADT) version 1.5.6 software.^50^ The CIF format files were converted to PDB files using mercury software. The 3D structure of the SARS-CoV-2 main protease (M^pro^) (PDB ID: 6Y2F),^51^ SARS-CoV-2 spike protein (PDB ID: 6M0J),^52^ duplex DNA (PDB ID: 1BNA),^53^ were obtained from the Protein Data Bank.^54^ Following the removal of water molecules, biomolecules and the compound were prepared in pdbqt format. Polar hydrogens and Gasteiger charges were added to the receptor. For 6Y2F and 6M0J, grid box sizes of 60 × 60 × 60 and 70 × 70 × 70 points in *x*, *y*, and *z* directions were used respectively, while blind docking was conducted for 1BNA. The grid space remained fixed at 3.75 Å, and 50 runs were performed utilizing Lamarckian genetic algorithm to identify optimal binding poses. Visualizing was done using Discovery studio^55^ and Pymol^56^ softwares.

## Results and discussion

**Scheme 1:**
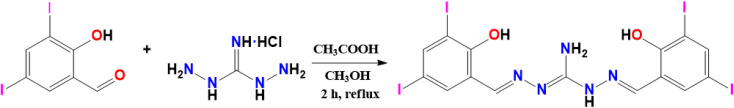
Schematic representation of the synthesis of the compound H_5_L.

The compound H_5_L was synthesized by the condensation reaction between 3,5-diiodosalicylaldehyde with 1,3-diaminoguanidine hydrochloride in methanol in the presence of a trace amount of acetic acid (Scheme 1). The compound was characterized by elemental analysis, MALDI-TOF MS, NMR, FT-IR and UV-visible solid state and solution phase spectra, while the molecular structure was confirmed by SCXRD. The compound was found soluble in DMSO and DMF, insoluble in water and some of the organic solvents like chloroform, methanol, acetonitrile, dichloromethane, ethyl acetate, *etc.* Though, the solution phase NMR results are indicating the expected symmetrical isomer the solid-state crystal structure is in agreement with its possible unsymmetrical isomer (Scheme S1†).

### Crystal structure of the proligand H_5_L

**Fig. 1:**
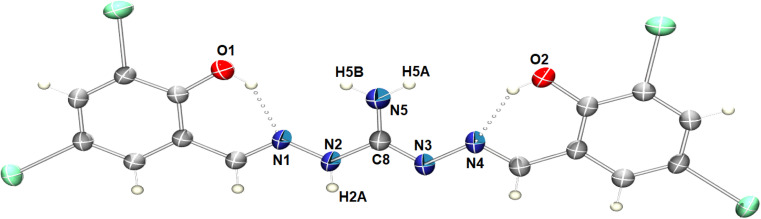
ORTEP diagram of H_5_L in 50% probability ellipsoids, showing intramolecular hydrogen bonds. DMSO solvent molecule is omitted for clarity.

**Fig. 2:**
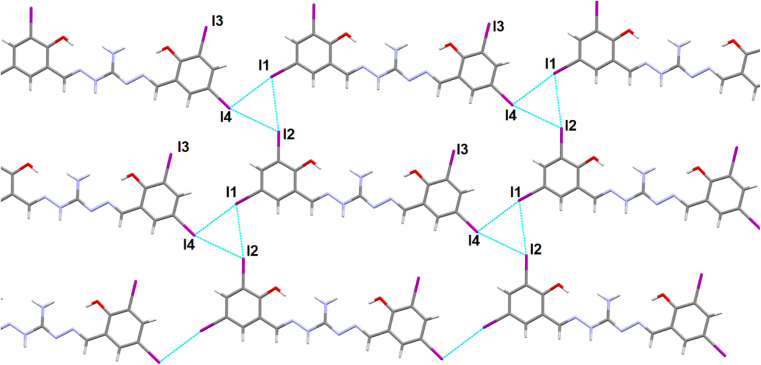
Formation of 2D network showing the triangular I_3_ halogen bonding interactions in the supramolecular network of the compound H_5_L. DMSO molecules are omitted for clarity.

Slow evaporation of the DMSO solution of the compound led to the formation of single crystals of the H_5_L. The crystal belongs to the monoclinic *P*2_1_/*m* space group and contain 2 molecules in each unit cell. Fig. 1 displays the molecular structure, highlighting intramolecular hydrogen bonding and the relevant numbering scheme. Table S2† presents relevant hydrogen bonding interactions, significant π⋯π interactions and a prominent iodine bonding interaction observed in the arrangement of the crystal lattice. Within the molecule, three conventional intramolecular hydrogen bonds occur between O1–H1A⋯N1, O2–H2⋯N4, and N2–H2A⋯O3, with respective hydrogen-acceptor distances of 1.88 Å, 1.89 Å, and 1.94 Å. Additionally, the oxygen atom of the DMSO solvent engages in weaker intermolecular hydrogen bonding with C16–H16C⋯O3, mutually complementing and playing a crucial role in the crystal packing.^57^ Moreover, phenylene rings of neighboring molecules participate in mutual π⋯π stacking interactions, with centroid–centroid distances measuring 3.5684(6) Å (Fig. S4†). The compound's intriguing feature involves four iodine bond interactions, playing a vital role in forming a 2D supramolecular triangular network interconnected through I1⋯I2, I1⋯I4, I1⋯I3 and I1⋯C13 (Fig. 2). The bifurcated nature of these interactions are leading to triangular I_3_ halogen bond connections in the network.

**Fig. 3:**
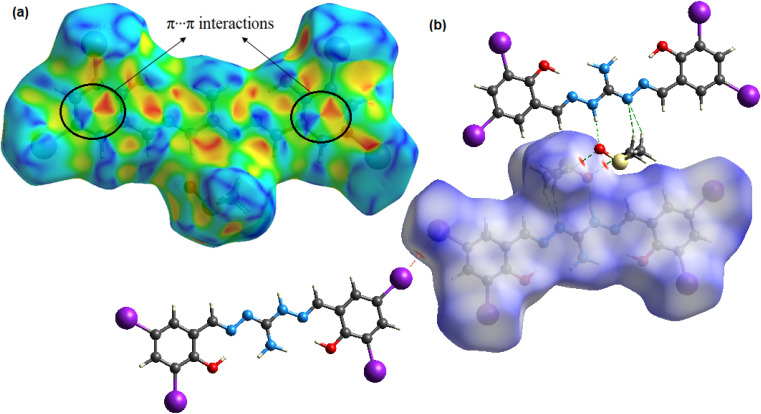
3D Hirshfeld maps with (a) shape index and (b) *d*_norm_ of the compound H_5_L.

Hirshfeld surface (HS) analysis quantifies intermolecular interactions in the crystal structure^58,59^ of the compound using crystal explorer 17.5 ^60^ software based on SCXRD data. The normalized contact distance function, *d*_norm_, combines distances to internal and external molecules on the HS. Color-coded regions represent van der Waals interactions, aiding in understanding crystal packing and structural properties.^58^ The compound's density normalization map (*d*_norm_) reveals highly intense red spots, which indicate the presence of intermolecular hydrogen bonding and iodine bonding regions. Additionally, the shape index function is utilized to examine the π⋯π stacking interactions within the crystal structure. The arrangement of red and blue triangles in the shape index map indicates that π⋯π interactions occur between the phenylene rings of the molecules. Fig. 3 displays the *d*_norm_ and shape index mappings, providing a visual representation of these findings. The 2D fingerprint plot of the compound provides valuable insights into the intermolecular interactions present in the crystal lattice.^61^ The plot shows the nature and type of these interactions, giving us a clear understanding of their relative contributions to the crystal packing. Among these interactions, H⋯I/I⋯H interactions hold the highest contribution (28.1% of HS area) and play a pivotal role in the crystal packing. Also, the significant relative contributions from H⋯H (18.1%), I⋯I (14.6%), and O⋯H/H⋯O (12.6%) interactions in the 2D fingerprint plot are indicative of the importance of iodine bonding interactions in the crystal lattice, which play a crucial role in reinforcing and stabilizing the crystal packing. Additionally, C⋯C interactions cover 9.3% of the respective HSs, indicating the presence of π⋯π stacking interactions. Furthermore, weaker interactions like C⋯H/H⋯C and N⋯H/H⋯N contacts contribute 6.5% and 2.9% to the HS area, respectively, further influencing the crystal packing (Fig. S5†).

### Spectral characteristics of the compound

The IR spectrum of the compound H_5_L displayed bands in the region of 1621 (DFT calculated 1650 cm^−1^) and 1674 cm^−1^ (calculated 1685 cm^−1^) attributed to *ν*(C=N) stretching vibrations. The *ν*(N–H) bands appeared at 3422 cm^−1^ (calculated 3533 cm^−1^) and a shoulder broad band at 3589 cm^−1^ (calculated 3645 cm^−1^) is assigned to *ν*(NH_2_) (Fig. S6†). Bands at 3222 and 3336 cm^−1^ (calculated 3239 and 3484 cm^−1^) indicate the presence of free *ν*(O–H). Additionally, bands observed at 1147 cm^−1^ (calculated 1256 cm^−1^) correspond to *ν*(N–N) and at 1347 cm^−1^ (calculated 1488 cm^−1^) attributed to *ν*(C–O) of the compound H_5_L. The DFT calculated vibrational frequencies are generally higher due to the absence of hydrogen bonding and non-covalent intermolecular interactions in the gas-phase optimization, compared to the experimental solid-state IR spectrum.^62^

The electronic spectrum of H_5_L (0.5 × 10^−4^ M) in solution phase was recorded using DMF solution. The compound exhibits bands at 475 (*ε* = 1780 M^−1^ cm^−1^) and 450 nm (*ε* = 5340 M^−1^ cm^−1^), which are assigned to *n* → π* transitions, while the bands observed at 272 (*ε* = 9932 M^−1^ cm^−1^), 334 (*ε* = 13 340 M^−1^ cm^−1^) and 382 nm (*ε* = 26 800 M^−1^ cm^−1^) are attributed to π → π* transitions (Fig. S7†). The solid-state electronic spectrum of H_5_L exhibits peaks at 282, 354, 368 and 418 nm corresponding to π → π* and *n* → π* transitions (Fig. S8a†). The band gap (*E*_g_) of the compound was experimentally determined by employing a Kubelka–Munk graph plotting (*F*(*R*)*hν*)^2^*versus* photon energy (*hν*), where *F*(*R*) represents the Kubelka–Munk function. The direct band gap energy was determined to be 3.08 eV (Fig. S8b†).

### Emission studies

**Fig. 4:**
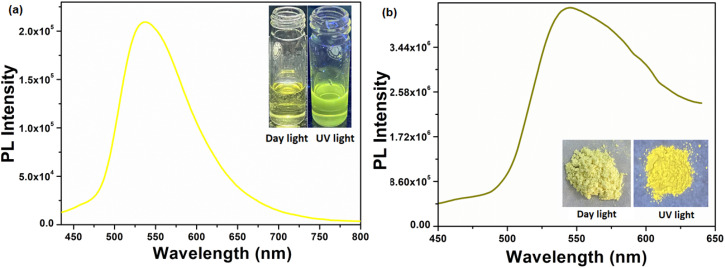
Solution phase (a) and solid state (b) emission spectra of the compound H_5_L (inset is the photographs under daylight and UV light).

The solution phase emission spectrum of the compound H_5_L was recorded using DMF. The compound was excited at a wavelength of 382 nm, and it displayed its highest emission intensity at 534 nm. In its solid state, the PL maximum of the compound was observed at 550 nm when excited at 354 nm. Notably, a vibrant yellow emission was observed with a redshift of 16 nm compared to its emission spectrum in the solution phase. Fig. 4 presents the emission spectra of the compound in both its solution phase and solid state, accompanied by photographs taken under daylight and UV light.

### Aggregation induced emission enhancement (AIEE) characteristics

**Fig. 5:**
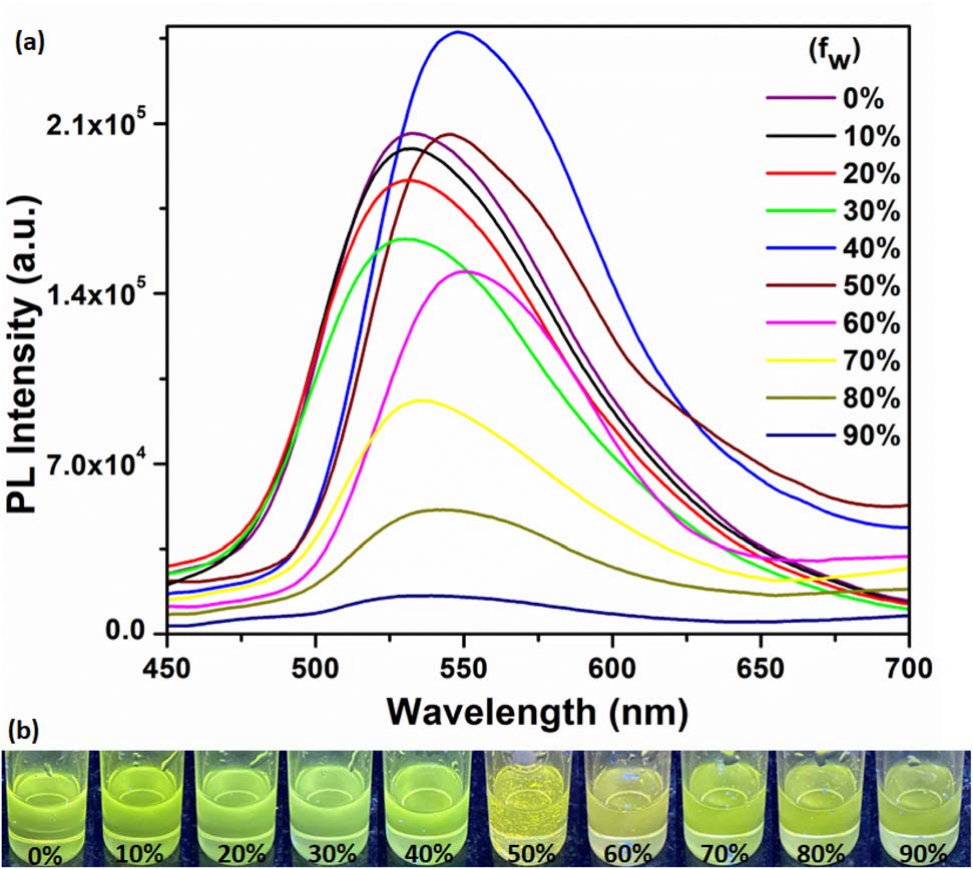
Fluorescence emission spectra of (a) H_5_L (excitation at 382 nm) in DMF/H_2_O binary mixture. The percentage represents the volumetric fraction (*f*_W_) of H_2_O, (b) optical images represent the AIEE of H_5_L under UV light (excitation at 345 nm).

Aggregation induced emission luminogens (AIEgens) are a class of materials that exhibit weak or no fluorescence when they are in a dissolved state in good solvents. However, when these AIEgens are aggregated or in the solid state, they display particularly strong fluorescence emission. The AIE effect occurs due to the restriction of intramolecular motion in the aggregated state, which prevents non-radiative pathways and enhances the radiative decay of excited states. Consequently, AIEgens emit bright fluorescence when they form aggregates or are in the solid state.^5,6^ In this study, we investigated the emission behavior of the compound by studying its aggregation properties in binary mixtures of DMF and water. Initially the compound was prepared in 10^−4^ M DMF solution. As the compound exhibits poor solubility in water, the introduction of water to DMF solution in different volumetric fractions results in molecular aggregation (Fig. 5a and S9†). The corresponding optical images depict the AIEE of the compound under UV light, as shown in Fig. 5b. Initially, the PL intensity decreased with the increase in water fraction until the *f*_W_ reaches 30%, indicating a notable quenching effect attributed to aggregation.^63^ However, an intriguing phenomenon was observed above *f*_W_ = 30%, there was a sudden increase in PL intensity at *f*_W_ = 40%, accompanied by a 16 nm redshift. The change in emission intensity is attributed to the AIE effect,^3,63^ mainly resulting from the restriction of intramolecular rotation mechanism^3,9^ and is associated with strong I⋯I type iodine bonding and strong O⋯H–N type hydrogen bonding interactions. Such non-covalent interactions are stronger than possible mutual π⋯π stacking interactions, thereby preventing non-radiative pathways and leading to radiative emission enhancement.^8^ The decreased PL intensity from 50% onwards could be attributed to increased aggregation behavior and strong intermolecular π⋯π stacking interactions, which diminish fluorescence emission.^3,64–66^ These features may be attributed to the dynamic interplay among various intermolecular forces and π⋯π stacking interactions upon aggregation. These findings provide valuable insights into the emission properties of the compound upon aggregation and indicate its potential usefulness in creating solid-state luminescent materials.

**Fig. 6:**
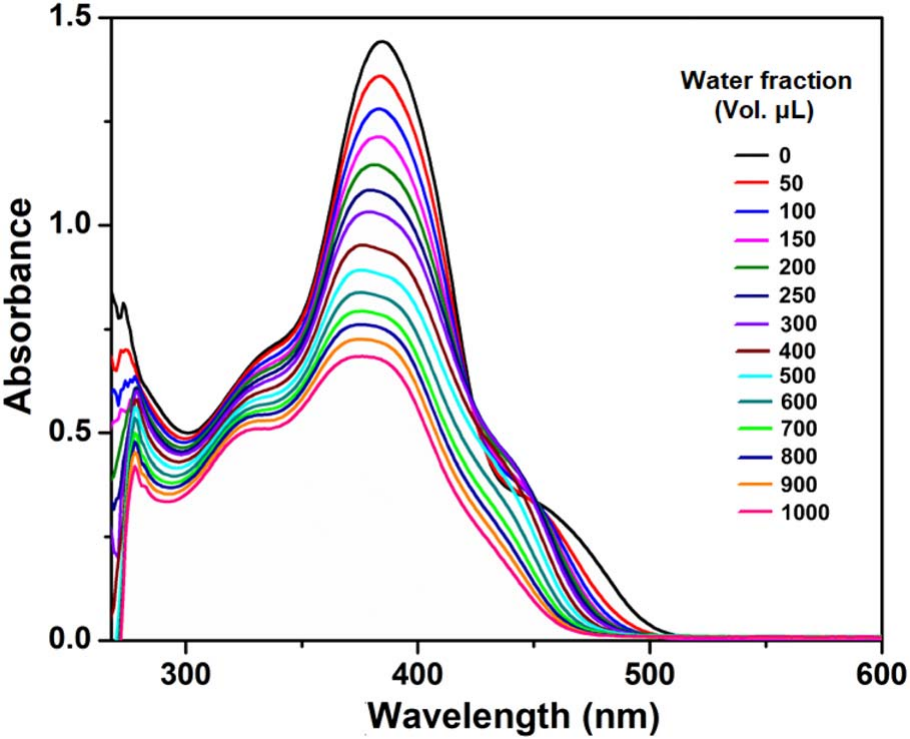
Absorption spectra of H_5_L in DMF/H_2_O binary mixture. The compound in DMF is taken as fixed (2000 μL) and added different volumetric fraction (*f*_W_) of H_2_O.

The UV-vis spectra of the compound (at a concentration of 50 μM) with varying water fractions were recorded and are depicted in Fig. 6. In dilute DMF solution, the compound H_5_L exhibits four major absorption peaks. As the water fraction is increased to (50–500 μL), the intensity of the peaks at around 475, 450, 382, 334 and 272 nm gradually diminishes, accompanied by a slight blueshift and broadening. This is attributed to the strong π⋯π stacking interactions between individual molecules during aggregation.^63,65^ Also, as the water fraction increases the intense band observed at 382 nm shows a splitting in the region of 370–382 nm, which might be attributed to oblique stacking rather than H-aggregate formation.

**Fig. 7:**
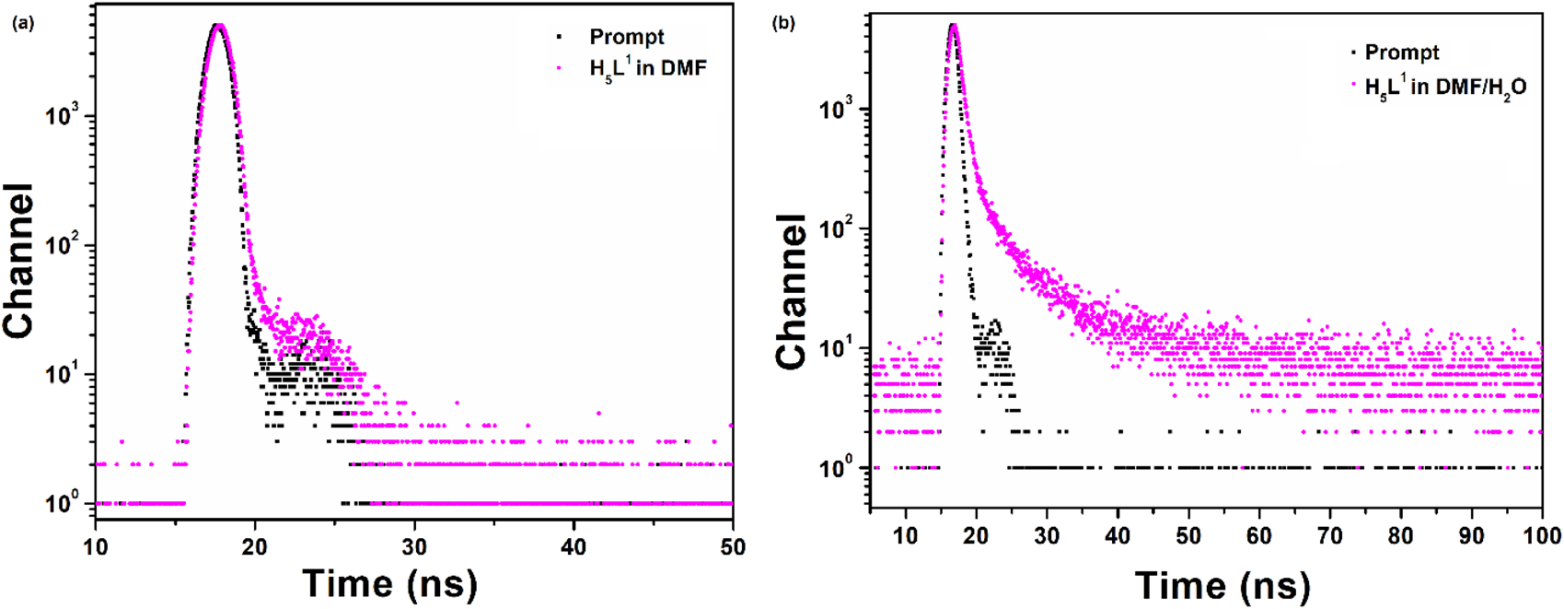
Time resolved fluorescence decay curves of the compound (a) in DMF and (b) in DMF/water (60/40%).

To gain a deeper understanding of the differences in photoluminescent properties between the solid-state and solution-phase, fluorescence lifetime investigations were conducted in both solution and aggregated states for the compound using the time-correlated single photon counting technique (TCSPC). The fluorescence decay of the compound in DMF only and water aggregates was analyzed using a biexponential expression, revealing average lifetimes of 0.15 ns and 0.91 ns respectively (Fig. 7). The fitting parameters are given in Table 2. The high lifetime value in the aggregated state, indicating the restricted intramolecular rotations of the compound. As a result, non-radiative pathways are obstructed, leading to increased excitation energy dissipation through radiative channels, leading to intense fluorescence.^44^ In the solution phase, unrestricted motion of the compound suppresses radiative decay, favoring nonradiative decay, resulting in low PL efficiency. However, in tightly constrained conditions, as in aggregation, spatial limitations block nonradiative decay, allowing radiative relaxation to exclusively drive high PL efficiency.^42,44,67^ In the analysis of lifetime decay and quantum yield in solution state (*Φ* = 0.020), it is apparent that the non-radiative decay constant (6.53 ns^−1^) significantly exceeds the radiative decay constant (0.133 ns^−1^). However, in the solid state (*Φ* = 0.360), the non-radiative decay constant decreases to 0.703 ns^−1^, while the radiative decay constant increases to 0.395 ns^−1^ due to aggregation. This observation suggests that the restricted intramolecular motion mechanism in the solid state, impeding the non-radiative decay process and thereby intensifying the fluorescence emission.

**Table 2:** Lifetime decay parameters of the compound

H_5_L	*τ*_1_ a (ns)	*τ*_2_ (ns)	*τ*_avg_ b (ns)	*χ* ^2^
DMF	0.15 (*α*_1_ = 99.9%)	2.33 (*α*_1_ = 0.1%)	0.15	1.00
Aggregate	5.71 ns (*α*_1_ = 2.3%)	0.79 ns (*α*_2_ = 97.7%)	0.91	1.51

a Fluorescence lifetime (% of fraction contribution to the emission profiles are given in parentheses).

b Mean lifetime.

### Theoretical study of the compound

**Fig. 8:**
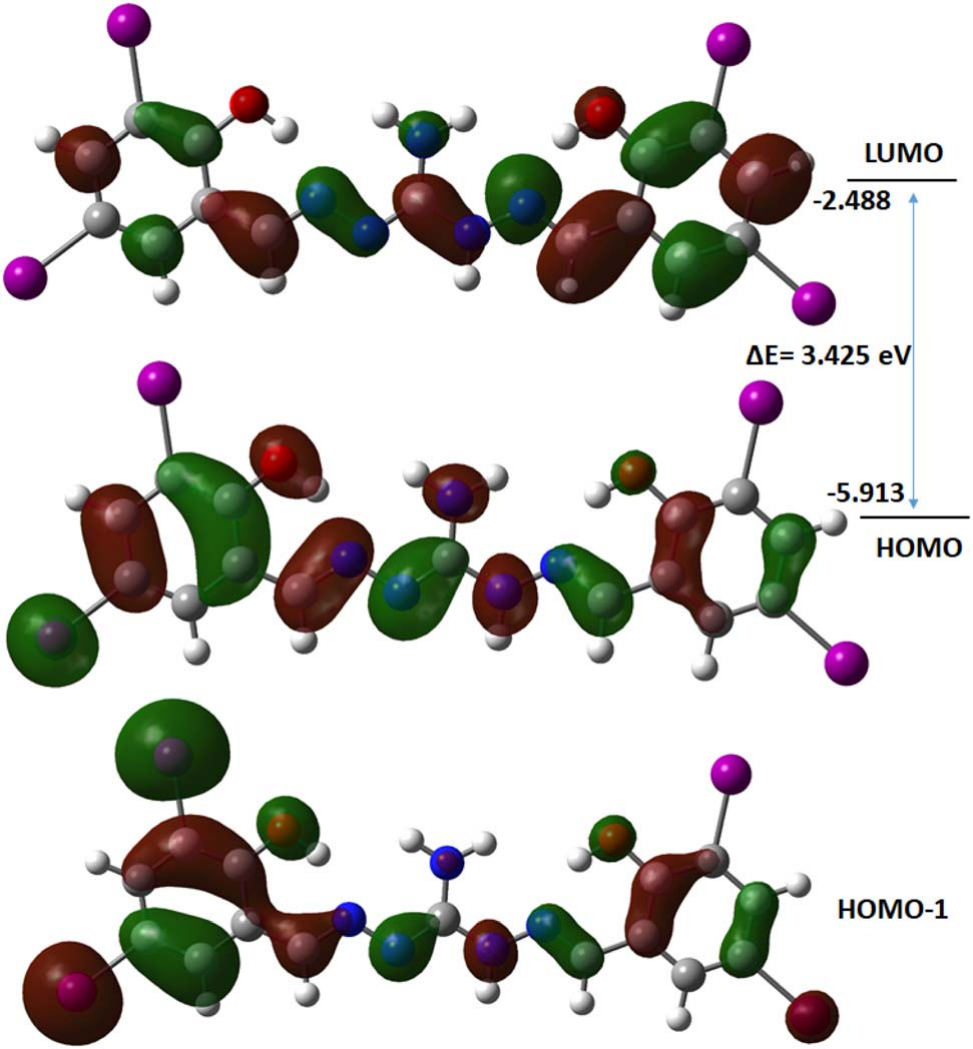
Frontier molecular orbitals of the compound.

Computational study was performed to gain insights into the spectral features and conduct FMO analysis. Theoretical investigations utilized the B3LYP/6-311G(d,p) and LanL2DZ basis set for obtaining optimized molecular structures. Chemical descriptors,^68^ including the hard-soft range of the compounds, were calculated using −*E*_HOMO_ as ionization energy and −*E*_LUMO_ as electron affinity, presented in Table S3.† The HOMO electron densities of the compound is found distributed over the free NH_2_ group and one of the iodine containing aminoguanidine moiety, while the LUMO electron densities are distributed over the aminoguanidine moiety other than NH_2_ and iodine. So, the possible HOMO to LUMO intramolecular charge transfer is mainly from iodine atoms to the phenylene moiety of the compound. The HOMO, LUMO, and neighboring orbitals of the compound exhibit negative energies, indicating molecular stability (Fig. 8). The compound is characterized by a higher negative chemical potential (*μ* = −4.200 eV) and moderate chemical hardness (*η* = 1.712 eV). In the gas phase, the FMO energy gap of H_5_L is calculated to be 3.425 eV, which closely agrees with the experimental band gap energy.

MEP maps visualize electron density distribution on molecular surfaces,^69^ aiding in predicting molecular shape, electrophilic and nucleophilic sites, and interactions between molecules.^70^ These maps are particularly valuable for gaining a comprehensive understanding of biological phenomena, including processes like enzyme–substrate binding, catalysis and drug-DNA interactions.^71^ In the compound, the blue color on the MEP maps indicates the presence of positive regions, while the red/yellow regions signify negative areas and green regions represent neutral areas within the compound. The phenolic oxygens of the compound display a faint red and yellowish color, indicating their susceptibility to electrophilic attack (Fig. S10†). Conversely, the blue regions represent electron-deficient areas, particularly mapped over the NH and free NH_2_, making them prone to nucleophilic interactions with proteins. Thus, the MEP maps provide valuable insights into the reactivity patterns and potential interaction sites of the compound with other molecules in biochemical processes.

The ADMET properties (absorption, distribution, metabolism, excretion, and toxicity) are critical in drug discovery. Drug failure often occurs due to safety and efficacy issues, so finding compounds with better ADMET properties is essential.^72^ In this study, the drug-likeness of the compound was assessed using *in silico* ADMET prediction with SwissADME^73^ and PreADMET^74^ online software. These analysis used to calculate the molecular weight (MW), number of rotatable bonds (*n*-ROTB), number of hydrogen bond donors (*n*-OHNH), number of hydrogen bond acceptors (*n*-ON), topological polar surface area (TPSA), the projected octanaol-water partition coefficient (log *P*_o/w_), the human intestinal absorption (HIA), cell permeability, Lipinski's violations and Veber's violation. Compared to cisplatin (HIA = 93.58%), the compound H_5_L is predicted to have a human intestinal absorption rate of more than 95% (Table 3). The cell permeability Caco-2 values are 19.88 for cisplatin and 15.16 for H_5_L indicating promising bioavailability. Additionally, H_5_L meets the drug-likeness criteria with acceptable values including log *P*_o/w_ (3.79), TPSA (115.59), *n*-OHNH (4) and *n*-ON (5), satisfying both Lipinski's and Veber's rules. These features strongly imply that the compound is well-positioned to demonstrate favorable bioavailability.^72,75^

**Table 3:** Physicochemical properties, lipophilicity and drug-likeness of the compound

Compounds	MW^a^	TPSA^b^	*n*-ROTB^c^	*n*-ON^d^	*n*-OHNH^e^	log *P*^f^	L.V.^g^	V.V.^h^	Caco-2^i^	HIA^j^
H_5_L	800.90	115.59	5	5	4	3.79	1	0	15.16	95.30
Cisplatin	300.05	6.48	0	2	6	—	1	0	19.88	93.58

a Molecular weight (≤500, expressed as g mol^−1^).

b Topological polar surface area (Å^2^).

c Number of rotatable bonds.

d Number of hydrogen bond acceptors (≤10).

e Number of hydrogen bond donors (≤5).

f Logarithm of partition coefficient (≤5) of compound between *n*-octanol and water.

g Lipinski's violations.

h Veber's violation.

i Caco-2 cell permeability (PCaco-2 (nm s^−1^), <4: low, 4–70: middle, >70: high).

j Human intestinal absorption, % (0–20 = poor, 20–70 = moderate, 70–100 = good).

### DNA binding study

**Fig. 9:**
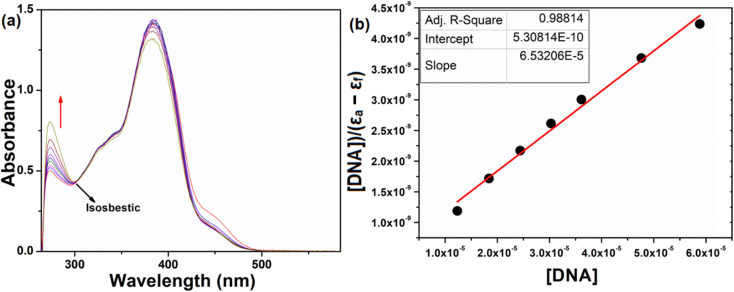
Absorption spectra of the compound (a) upon addition of CT-DNA {[compound] = 50 μM [DNA] = 12–70 μM}, (b) plot of DNA/(*ε*_a_ − *ε*_b_) *versus* [DNA] for the titration of H_5_L with DNA.

Electronic absorption spectroscopy plays a pivotal role in investigating DNA binding interactions. The study was carried out by gradually adding CT-DNA to a solution with a constant compound concentration (50 μM). As DNA concentrations were incremented within the range of 12 to 70 μM, distinct changes in the absorption characteristics of the compound were observed (Fig. 9). Specifically, the absorption band at 272 nm displayed hyperchromism, while the bands at 334, 382, 450 and 475 nm exhibited hypochromism. An intriguing finding was the emergence of an isosbestic point at 300 nm. This point is indicative of a significant change in the system, suggesting that a distinct chemical process is taking place. The presence of an isosbestic point reinforces the idea that a strong interaction occurs between the DNA and the compound. The intrinsic binding constants (*K*_b_) for the interaction was determined as 1.23 × 10^5^ M^−1^ using eqn (4). The absorbance intensity variation with no redshift observed for the compound, indicating a groove mode of binding with DNA.^76,77^ In essence, this set of observations supports the conclusion that the compound and DNA are engaging in a substantial and meaningful interaction.

**Fig. 10:**
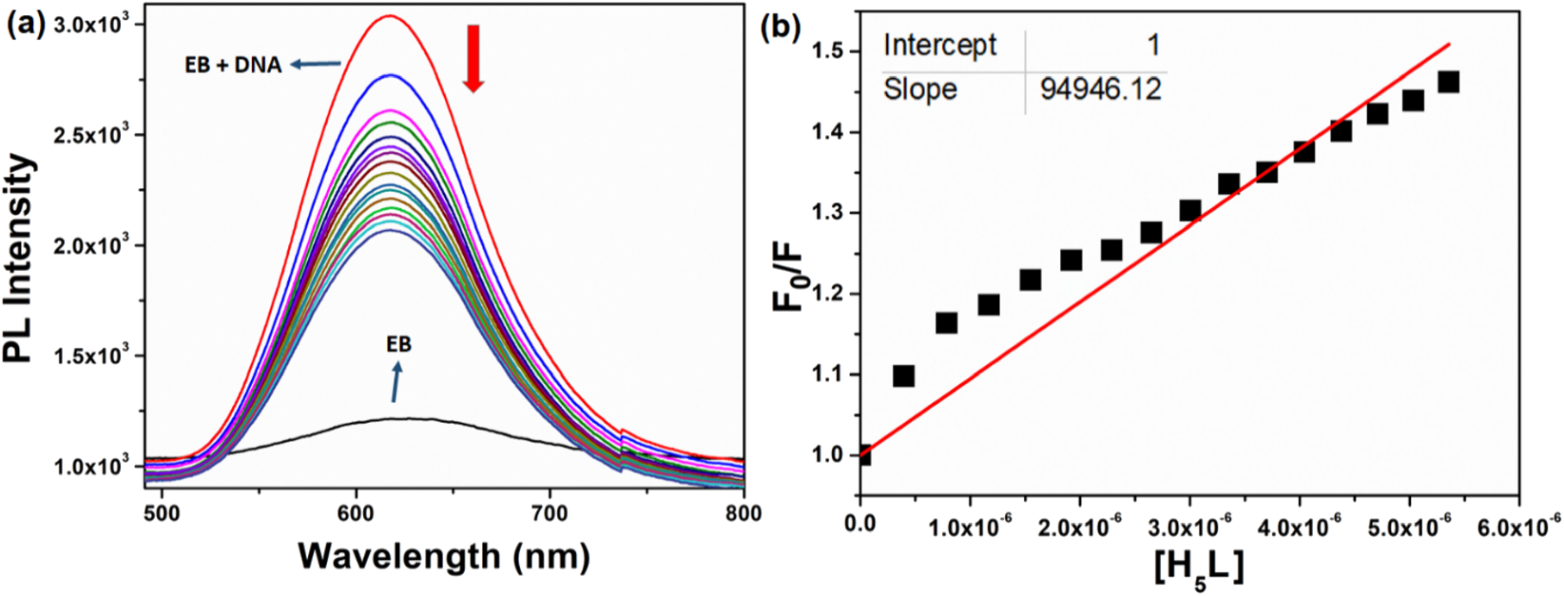
(a) Emission spectrum of EB bound to DNA with different concentration of the compound. The red arrow shows the decrease in intensity upon the increase of the compound concentrations. (b) Plots of *F*_0_/*F versus* [H_5_L] for the titration of H_5_L with DNA.

Fluorescence is a significant spectroscopic method employed to investigate the interaction between bioactive compounds and biomolecules. EB serves as a fluorescence probe, intensifying DNA fluorescence when intercalated into its base pairs.^78^ Incremental compound addition to EB-treated DNA induces fluorescence quenching, implying EB replacement by the compound. The experiment highlights substantial fluorescence quenching of CT-DNA-EB due to gradual compound addition (0.3–5 μM) (Fig. 10). The quenching constant (*K*_q_) for the compound was measured as 9.4 × 10^4^ M^−1^. A higher *K*_q_ can suggest a stronger interaction, which might be due to factors like binding affinity or proximity between the two molecules, suggesting its potential utility as a drug or probe in biological assays.

**Fig. 11:**
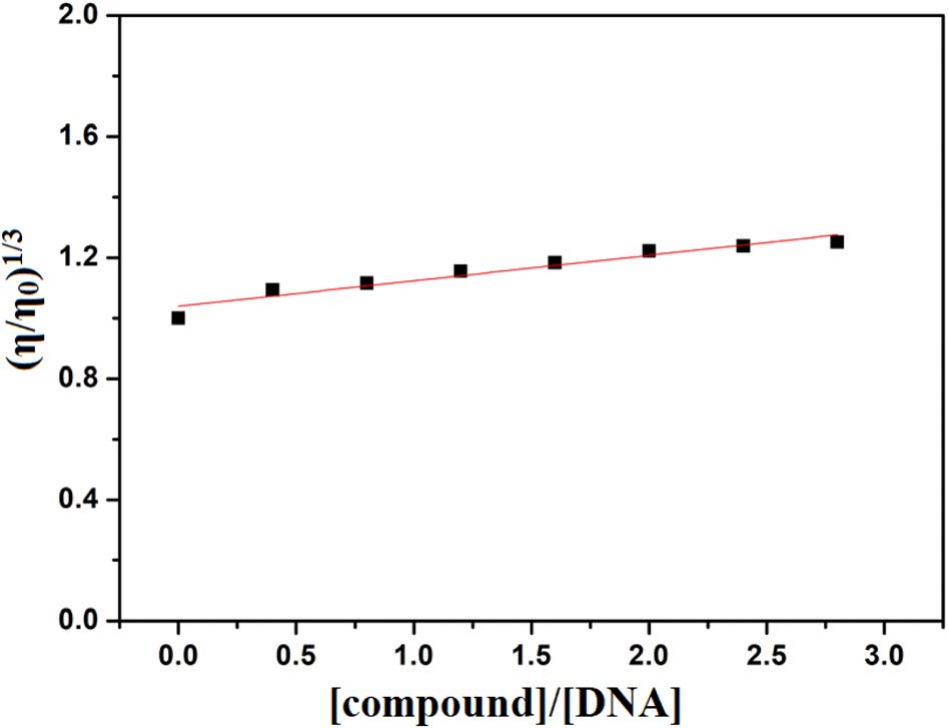
Effect of increasing concentration of H_5_L on the relative viscosity of CT-DNA.

To understand the interaction between the compound and DNA, viscosity measurements were also performed. When small molecules intercalate into DNA, they separate the base pairs, elongating the DNA and resulting in a significant increase in viscosity.^79^ In contrast, groove binding typically make minimal effects on DNA length and therefore have partial or no change in the viscosity of the DNA solution.^77,80^ Here, the gradual addition of the compound to CT-DNA resulted in a relatively minor change in viscosity, indicating its role as a groove binder (Fig. 11).^76^

### *In vitro* study

*In vitro* cytotoxicity studies hold pivotal significance in cancer drug development by evaluating a compound's potential to selectively kill cancer cells, guiding the selection of promising candidates for effective anticancer therapies. Here, the cytotoxicity on human breast cancerous cell MCF-7 was found to be increasing as the drug concentration increases. The estimated IC_50_ value (181.05 μg mL^−1^) indicates that the compound demonstrates superior activity (Table S4†). The lesser toxicity observed against the L929 normal fibroblast cells, with an IC_50_ value of 356.54 μg mL^−1^, is also promising. However, *in vivo* studies are also required to validate its potential as a cytotoxic agent against breast cancer cells. The phase-contrast images and the cytotoxicity effect of the compound on MCF-7 and L929 cells at different concentrations are given in Fig. S11–S13.†

### Molecular docking study

Utilizing molecular docking, to simulate and predict the binding interactions between small drug-like molecules and target biomolecules, facilitates the identification of potential lead compounds and accelerating the drug discovery process. The docking studies were conducted to predict the binding modes of H_5_L with the duplex DNA, the active sites of the SARS-CoV-2 M^pro^ and SARS-CoV-2 spike protein. Table 4 provides the details on docking energy, inhibition constants, relevant hydrogen bonding, and hydrophobic/electrostatic interactions between the compound and the targeted biomolecules.

**Table 4:** Docking energy and interactions of the compound with duplex DNA and SARS-CoV-2

Biomolecule	Docking energy (kcal mol^−1^)	Inhibition constant, *K*_i_ (nM)	Interaction with residues	
			Hydrogen bonding	Residues involving hydrophobic/halogen and other interactions
1BNA	−13.72	0.087	O1(H1A): DT7(O2)	
			O2(H2): DG10(O4′)	DA18(C2): O1
			N5(H5B): DC9(O3′)	C7: DA18(N3)
			N5(H5A): DC9(O3′)	C7: DT8(O2)
			DG16 (N2): O2	DG16: I3
			DG10 (N2): I3	DC11: I3
6Y2F	−9.57	96.02		THR190(O): I3
				CYS44(O): I2
			O2(H2): GLU166(O)	THR26(O): I1
			CYS145(SH): N1	Phe1: CYS145
			N2(H2A): HIS164(O)	Phe2: MET165
				I1: LEU27
				I4: MET165
6M0J	−9.25	165.05		LYS187: Phe1
			ASP206(N): I3	ASP509: Phe1
			SER511(N): N4	TRP203: Phe2
			ARG514(NH1): N4	I1: LEU120
			N5(H5B): ASP509(O)	I2: LEU120
			O2(H2): GLU398(OE1)	TYR202: I4
			O1(H1A): ASN508(O)	TRP203: I4
			O1(H1A): ASP509(O)	Phe1: LEU120

### Docking with duplex DNA

**Fig. 12:**
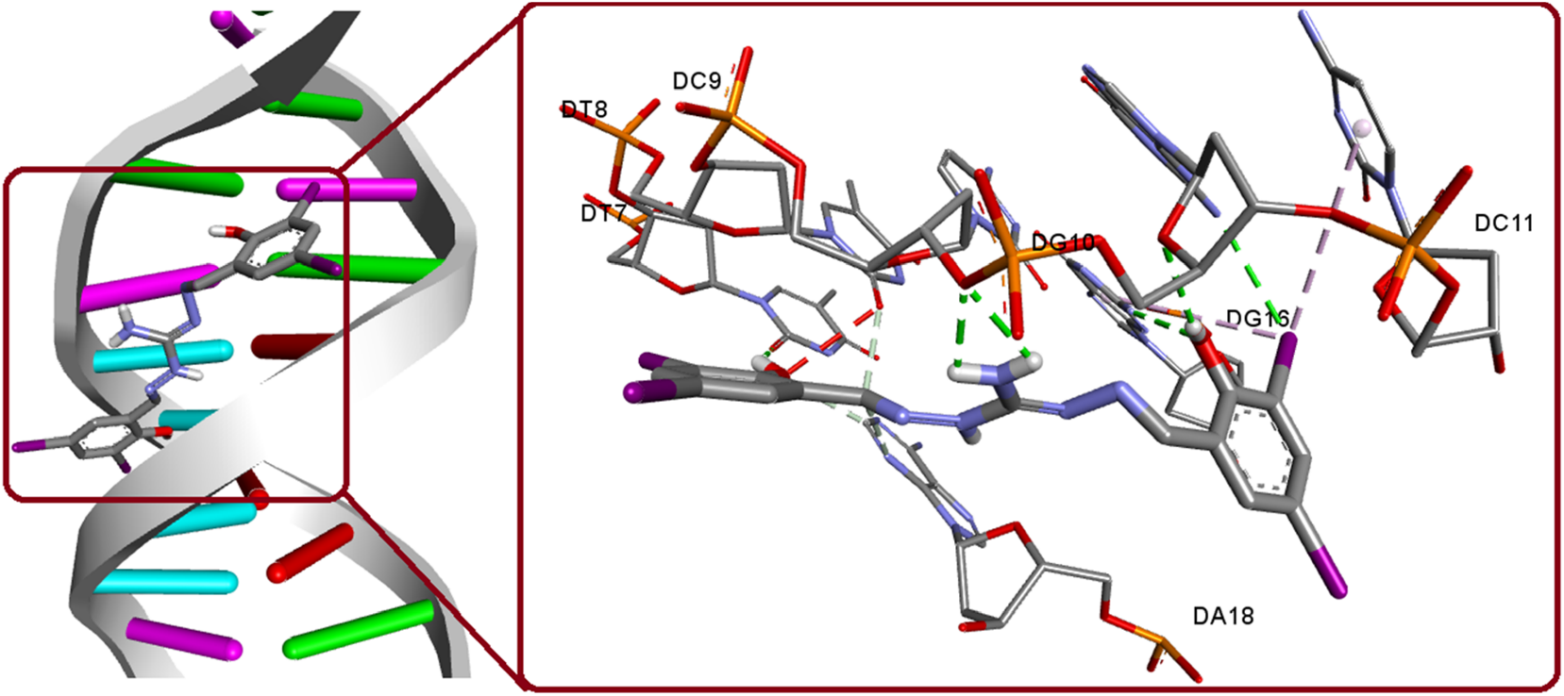
Binding modes of H_5_L and its focused view of interactions with 1BNA.

The interaction between DNA and potential anticancer compounds holds significant biological relevance as DNA plays a vital role in many essential cellular mechanisms and making it a prime target for anticancer agents. By making use of the crystal structure of 1BNA from the PDB, at a resolution of 1.9 Å, docking experiments were conducted to unveil the possible interaction. The docking study findings, with a binding score of −13.72 kcal mol^−1^ along with its groove mode of binding, are consistent with the experimental results, which strengthens the compound's potential as an anticancer agent. The compound exhibit strong hydrogen bonding interactions with specific DNA regions involving O1(H1A)⋯DT7(O2), O2(H2)⋯DG10(O4′), N5(H5B)⋯DC9(O3′), N5(H5A)⋯DC9(O3′), DG16(N2)⋯O2. Also, a relevant hydrogen bonding observed as hydrogen donor from DG10(N2) and iodine I3 as acceptor (Fig. 12). The significance of iodine in bonding interaction extends to its biological relevance, where iodine's unique properties such as its large size and polarizability influence its interactions with biomolecules. Targeting DNA with potential anticancer agents can disrupt cancer cell growth and proliferation, making this study's insights essential for advancing cancer therapeutics. The docking simulations with experimental validation provide valuable groundwork for future research in developing effective and targeted cancer treatments.

### Docking with SARS-CoV-2 M^pro^

**Fig. 13:**
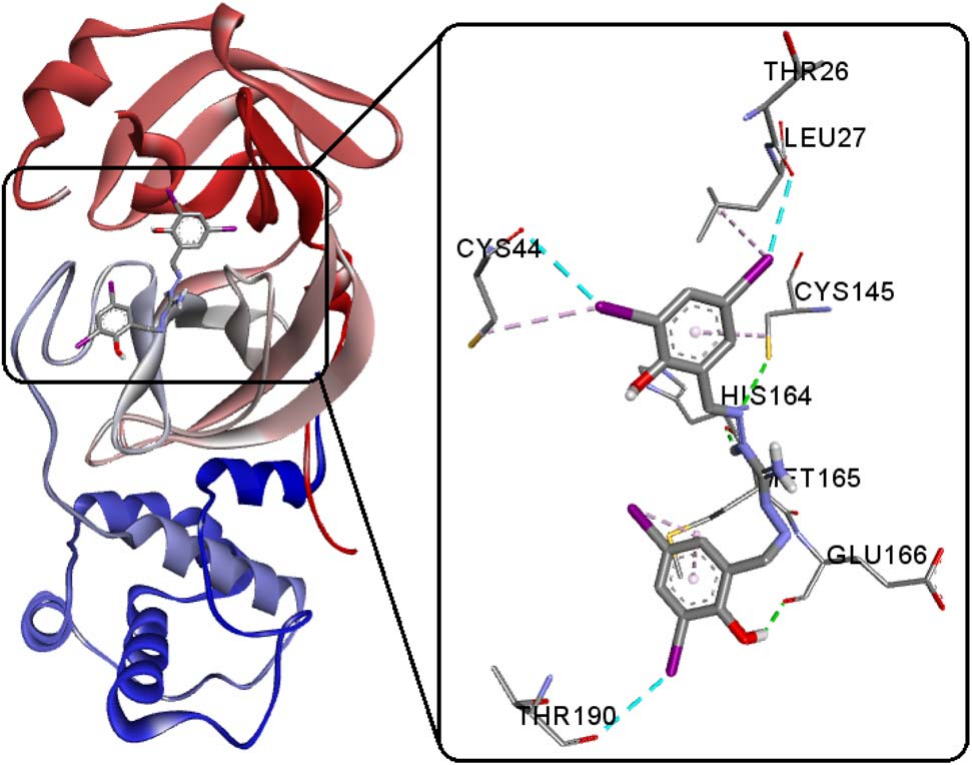
Interactions of H_5_L and its focused view at the active site of SARS-CoV-2 M^pro^.

SARS-CoV-2, classified within the β-coronavirus family, exhibits proteins like the M^pro^ or 3CL^pro^, presenting promising drug targets for impeding viral replication.^81,82^ These proteins play a key role in the virus's life cycle, as they facilitate the proteolytic processing of viral polyproteins, essential for viral maturation and infectivity.^81,83–85^ Targeting M^pro^ or 3CL^pro^ with therapeutic interventions can hinder the virus's ability to replicate and propagate, thus offering a potential avenue for antiviral drug development against COVID-19. The inhibition of these crucial proteins holds substantial promise in controlling the progression of the disease and mitigating its impact on global public health. In our study, we performed comparative docking calculations for the new compound H_5_L (−9.57 kcal mol^−1^) with its recently reported oxygen analogous luminogen bis(3,5-diiodosalicylidene)carbohydrazone (H_4_L^1^, −7.80 kcal mol^−1^) and its sulfur counterpart bis(3,5-diiodosalicylidene)thiocarbohydrazone (H_4_L^2^, −9.06 kcal mol^−1^).^8^ This signifies a stronger binding affinity, suggesting that H_5_L possesses a more favorable interaction with the target including three conventional hydrogen bonding between GLU166(O)⋯O2(H2), HIS164(O)⋯N2(H2A) and CYS145(SH)⋯N1 (Fig. 13 and S14†). Furthermore, the compound H_5_L demonstrates three robust I⋯O halogen bonding interactions with key active site residues THR26(O), CYS44(O), and THR190(O). This strong and concise I⋯O interaction of H_5_L potentially reinforces the binding between the compound and the enzyme. Such interactions offer insights into biomolecular drug design and engineering strategies, highlighting the potential of halogen bonds as a tool for enhancing ligand–protein interactions.^86–88^ Importantly, the compound exhibit better binding energy than that of co-crystal (−8.90 kcal mol^−1^) chloroquine (CQ) (−6.38 kcal mol^−1^), hydroxychloroquine (HCQ) (−6.69 kcal mol^−1^) along with the repurposed drugs lopinavir (−8.10 kcal mol^−1^), remdesivir (−8.32 kcal mol^−1^), darunavir (−8.67 kcal mol^−1^) and ritonavir (−8.97 kcal mol^−1^) that are specifically active at 6Y2F,^8,89^ indicating its potential as a potent candidate for targeted molecular interactions.

### Docking of the luminogen with SARS-CoV-2 spike protein

**Fig. 14:**
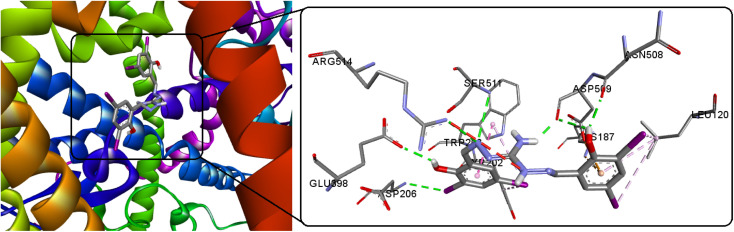
Docked conformations of H_5_L and its focused view at the active site of SARS-CoV-2 spike protein.

The life cycle of SARS-CoV-2 initiates with its entry into human cells, facilitated by the virus's spike protein. This protein, present on the virus surface, interacts with the angiotensin-converting enzyme receptor-2 (ACE-2). Consequently, the spike protein emerges as a significant target for developing both SARS vaccines and anti-viral medications. In the pursuit of specific infectious agent detection, including viruses, AIE-based systems and modified AIEgens serve as valuable bioprobes. These systems enable precise identification and targeting of infectious agents. Utilizing AIEgens as bioprobes offers a promising approach for enhancing ability to detect and combat various pathogens, including viruses, and contributes to advancing diagnostic and therapeutic strategies in the realm of infectious diseases. The study encompassed virtual screening against the SARS-CoV-2 spike glycoprotein (PDB ID: 6M0J, chain A) to discern the most advantageous binding configuration for the fluorescent molecule. This approach aimed to gain insights into the specific interaction pattern that would facilitate effective binding. The outcomes of molecular docking computations highlight a favorable interaction with the spike glycoprotein, as demonstrated by a docking energy of −9.25 kcal mol^−1^ and the formation of seven potent hydrogen bonds, signifying a promising binding affinity (Fig. 14 and S15†). In comparison, the new compound H_5_L exhibits superior binding effectiveness compared to its recently reported (thio)carbohydrazone luminogens (H_4_L^1^ = −7.34, H_4_L^2^ = −5.88 kcal mol^−1^).^8^ This information could contribute significantly to the design and development of potential antiviral agents or diagnostic tools targeting the SARS-CoV-2 spike glycoprotein.

## Conclusion

A new bioactive and luminogen proligand derived from aminoguanidine is reported. SCXRD and HS analysis reveals prominent triangular iodine bonding, along with various interactions, influencing the crystal lattice packing. Remarkably, the multidentate compound H_5_L exhibited fluorescence in both solid and solution phases and showcasing AIEE characteristics. This phenomenon was further confirmed through fluorescence lifetime decay investigations. Experimental DNA binding studies, employing fluorescence, absorption and viscosity studies, revealed the compound's affinity *via* groove binding, with a binding constant (*K*_b_) of 1.23 × 10^5^ M^−1^ and a quenching rate constant (*K*_q_) of 9.4 × 10^4^ M^−1^. This was corroborated by *in silico* molecular docking, which yielded a favorable binding energy of −13.72 kcal mol^−1^ and underscoring the strong interaction with CT-DNA. The compound showed lesser toxicity in normal cell lines during *in vitro* cytotoxicity assessment, while the MCF-7 human breast cancer cell line demonstrated notable efficacy of H_5_L, with an IC_50_ value of 181.05 μg mL^−1^, suggesting its potential as an effective anticancer agent. Additionally, the compound displayed significant binding capability with the main protease 3CL^pro^ of SARS-CoV-2 (−9.57 kcal mol^−1^), surpassing several conventional and specifically active repurposed drugs such as CQ, HCQ, lopinavir, ritonavir, darunavir, and remdesivir, indicating its potential as a prospective candidate for combating COVID 19. Moreover, H_5_L exhibited favorable binding affinities towards the SARS-CoV-2 spike protein, with an exceptional binding energy of −9.25 kcal mol^−1^, suggesting its potential utility as a bioprobe for the analysis of SARS-CoV-2 and related biological processes. Given the critical role of luminescent bioprobes in enhancing detection sensitivity in nucleic acid and immunological assays, this novel aminoguanidine-derived luminogen would open up exciting possibilities for monitoring and studying the apoptotic mechanisms of either breast cancer or SARS-CoV-2 and associated biological phenomena.

## Author contributions

K. K. Mohammed Hashim: conceptualization, data curation, formal analysis, investigation, methodology, software, visualization, writing – original draft. E. Manoj: conceptualization, investigation, funding acquisition, validation, project administration, resources, software, supervision, validation, visualization, writing – review & editing.

## Conflicts of interest

The authors declare no conflict of interest.

## Supplementary Material

RA-014-D4RA00554F-s001

RA-014-D4RA00554F-s002
