# Supplementary material for: Aminoguanidine-based bioactive proligand as AIEE probe for anticancer and anticovid studies
Source: RSC Adv. 2024 Apr 25;14(19):13654–68. doi: 10.1039/d4ra00554f (PMC11044126; doi:10.1039/d4ra00554f)
Supplement: RA-014-D4RA00554F-s001 [file RA-014-D4RA00554F-s001.pdf]

Electronic Supplementary Material (ESI) for RSC Advances.
This journal is © The Royal Society of Chemistry 2024

Supplementary Information for

Aminoguanidine-Based Bioactive Proligand as AIEE Probe for Anticancer and Anticovid Studies

K. K. Mohammed Hashim[a], E. Manoj[a*]

Sl. No.	Contents	Page no.
1	**Scheme S1**. The two possible isomers of H_5L in solution (left) and solid (right) phase.	S3
2	**Figure S1.** ^{1}H-NMR spectrum of H_5L in DMSO-d_6.	S3
3	**Figure S2.** ^{13}C-NMR spectrum of H_5L.	S4
4	**Figure S3.** MALDI-MS spectrum of H_5L.	S4
5	**Figure S4.** Packing diagram of H_5L showing hydrogen bonding (Blue) and π ··· π stacking (Green) interactions along the a-axis.	S5
6	**Figure S5.** 2D fingerprint plots of H_5L showing the percentage contacts contributing to the total Hirshfeld surface area of the molecule.	S6
7	**Figure S6.** Experimental (a) and theoretical (b) IR spectra of H_5L.	S6
8	**Figure S7.** Solution state UV-vis spectrum of H_5L.	S7
9	**Figure S8.** Solid state (a) UV-vis spectrum and (b) band gap energy calculations of H_5L from Kubelka-Munk plot.	S7
10	**Figure S9.** Fluorescence emission intensity plot of H_5L in DMF/H_2O binary mixture.	S8
11	**Figure S10**. Molecular electrostatic potential maps of the compound.	S8
12	**Figure S11**. Phase-contrast images of the compound on MCF-7 at different concentrations in μg/mL.	S9

* Corresponding author.
E-mail address: manoje@cusat.ac.in (E. Manoj).

13	**Figure S12**: Phase-contrast images of the compound on L929 at different concentrations in μg/mL.	S10
14	**Figure S13**: Cytotoxicity effect of H_5L on MCF-7 (left) and L929 cell line (right).	S10
15	**Figure S14**. 2D representation of the compound with 6Y2F.	S11
16	**Figure S15**. 2D representation of the compound with 6M0J.	S11
17	**Table S1.** Selected bond lengths (Å) and bond angles (˚) of H_5L.	S12
18	**Table S2.** Interaction parameters of the compound H_5L.	S13
19	**Table S3.** The calculated chemical reactivity parameters for the compound (The energy parameters were calculated by assuming $-E_{HOMO}$ as ionization energy and $-E_{LUMO}$ as electron affinity).	S14
20	**Table S4.** Percentage viability of MCF-7 and L929 cells with concentration of the proligand.	S14

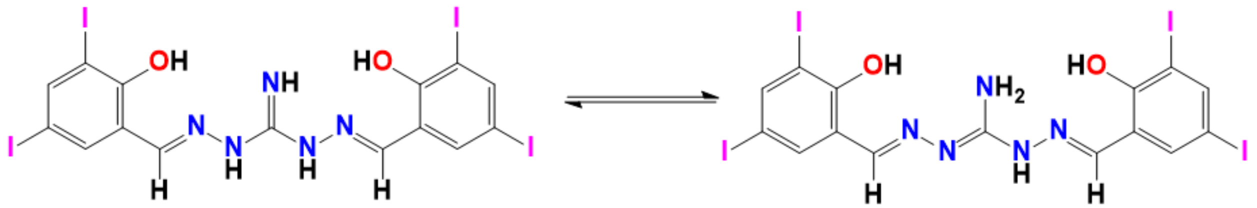

Scheme S1. The two possible isomers of H_5L in solution (left) and solid (right) phase.

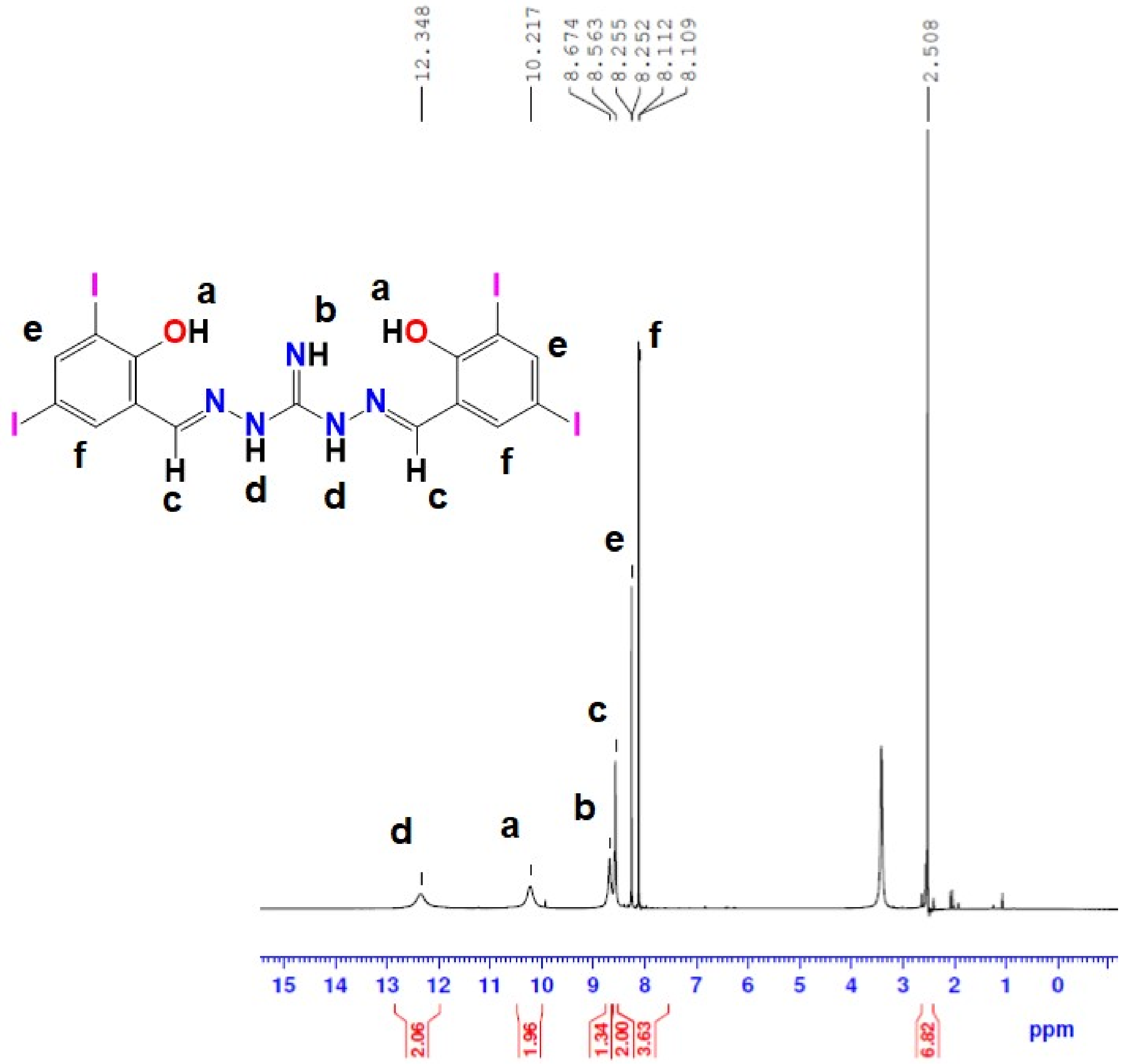

Figure S1. ^{1}H-NMR spectrum of H_5L in DMSO-d_6.

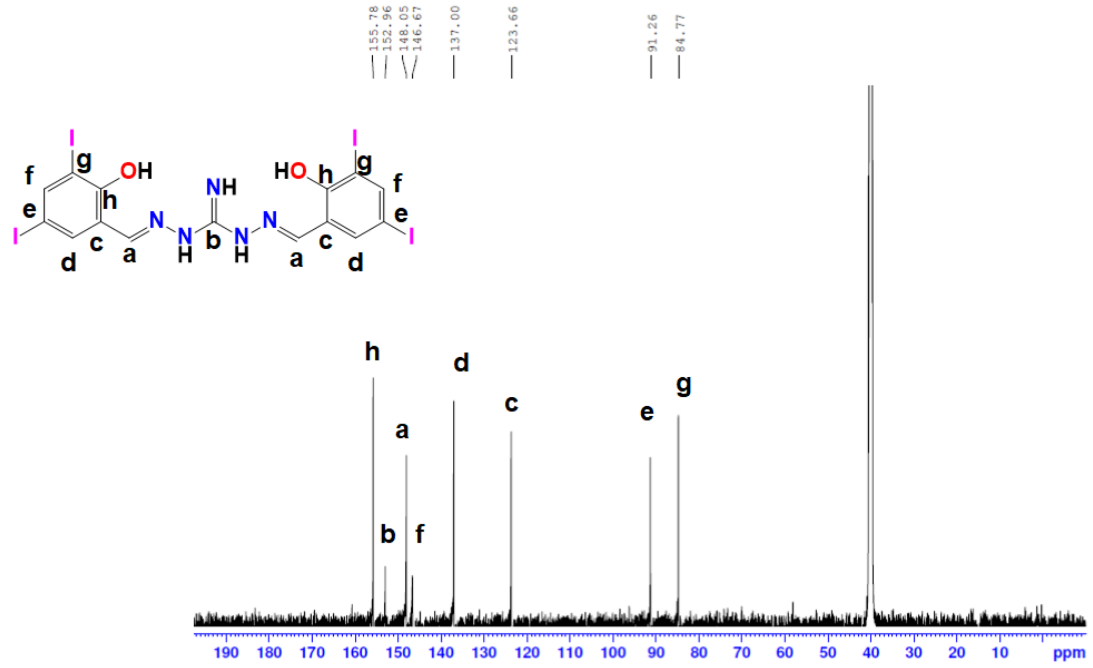

Figure S2. ^{13}C-NMR spectrum of H_5L.

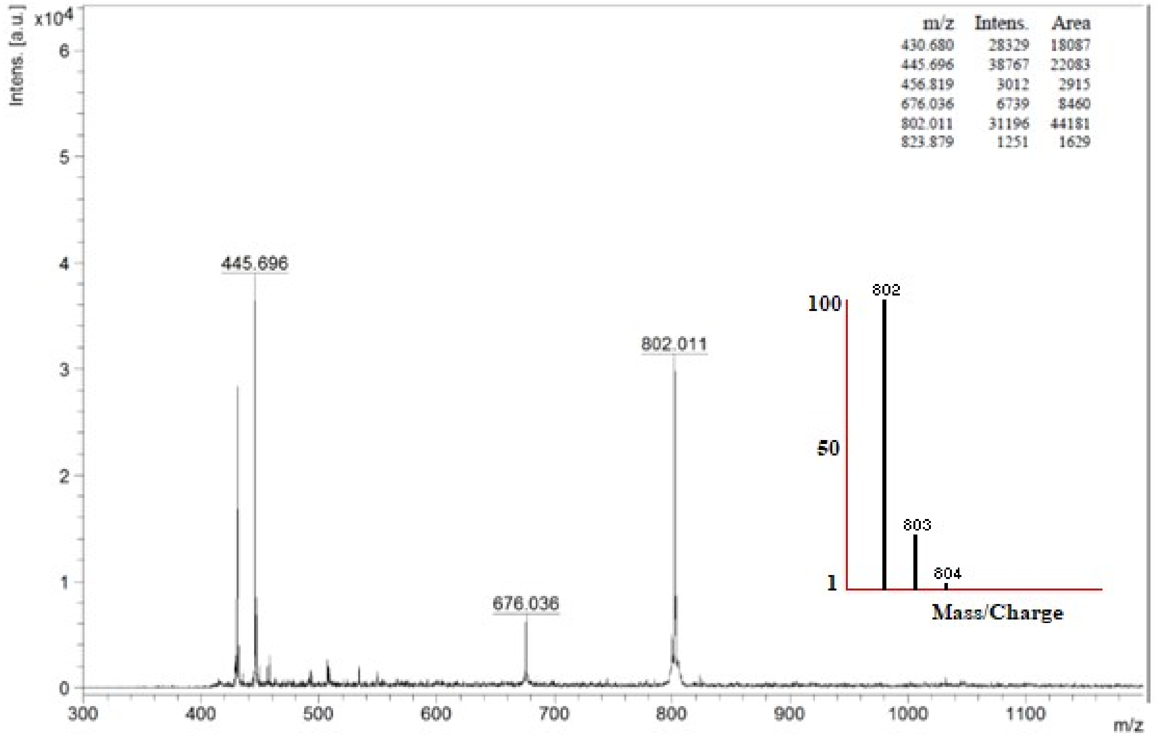

Figure S3. MALDI-MS spectrum of H_5L. The insert showing the calculated isotopic pattern of the molecular ion peak.

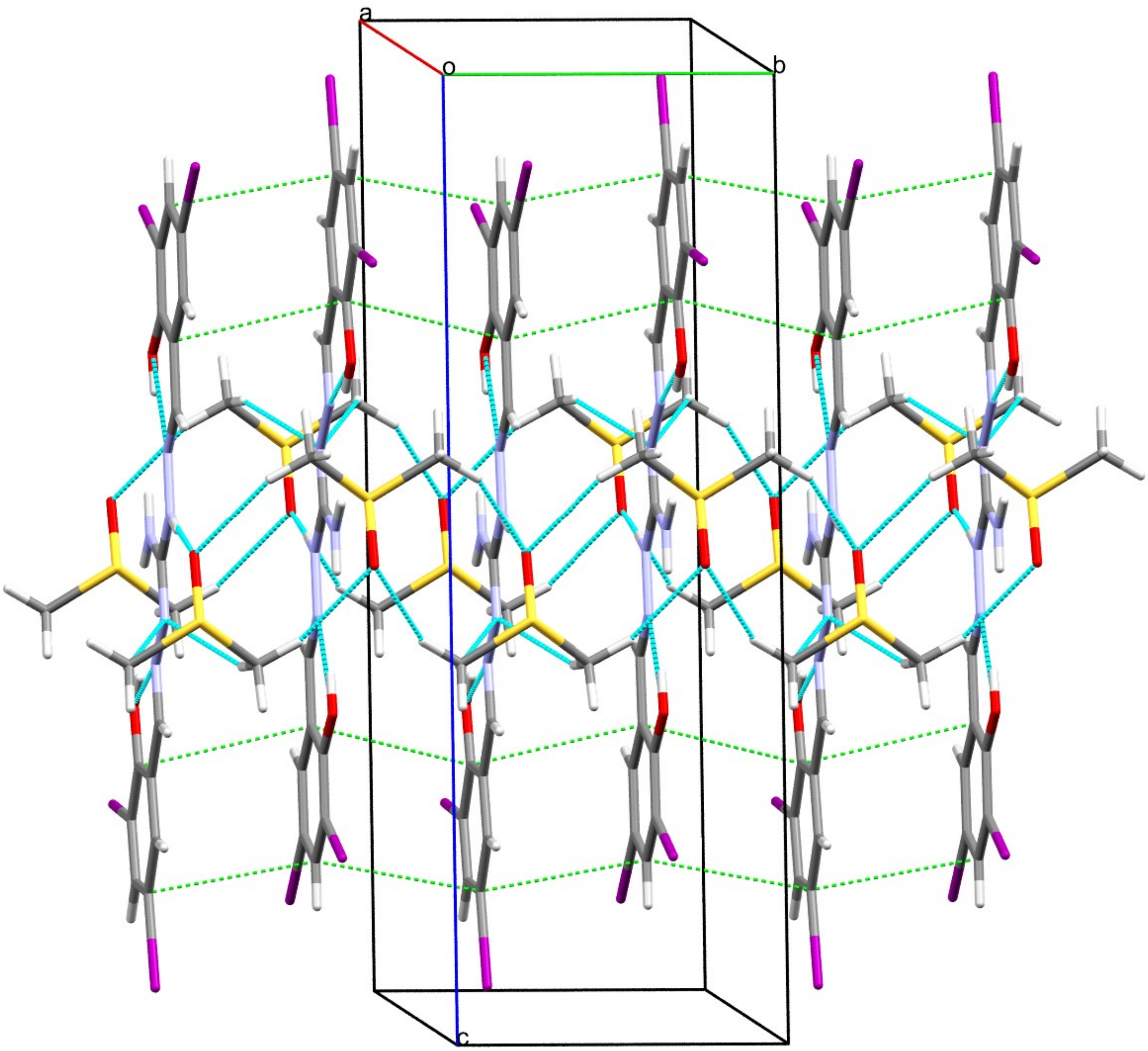

Figure S4. Packing diagram of H_5L showing hydrogen bonding (Blue) and π ··· π stacking (Green) interactions along the a-axis.

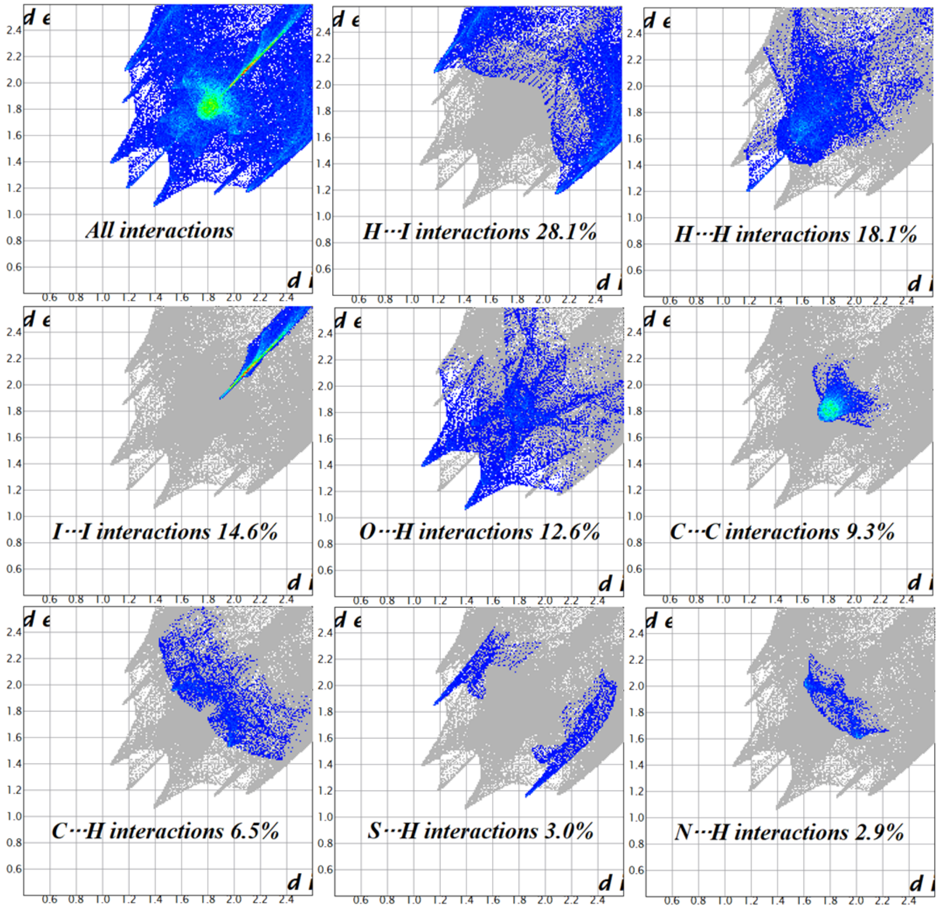

Figure S5. 2D fingerprint plots of H_5L showing the percentage contacts contributing to the total Hirshfeld surface area of the molecule.

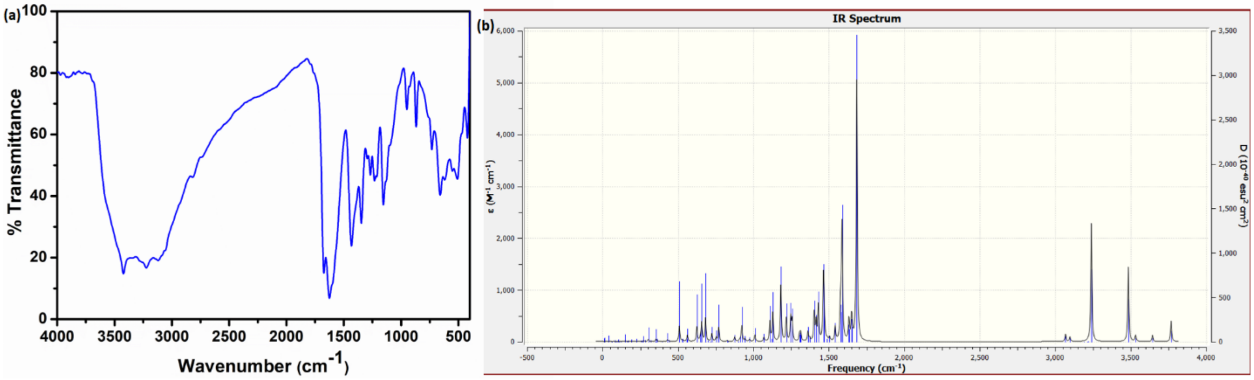

Figure S6. Experimental (a) and theoretical (b) IR spectra of H_5L.

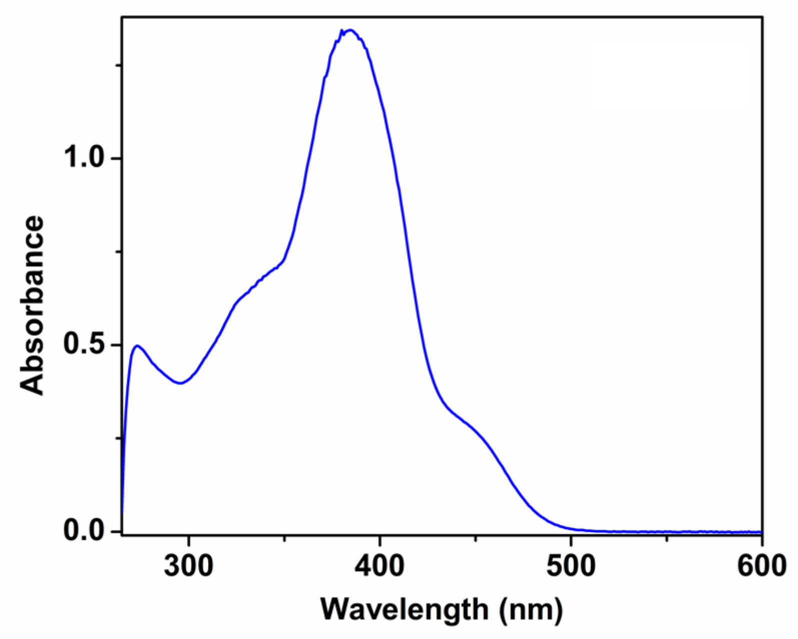

Figure S7. Solution state UV-vis spectrum of H_5L.

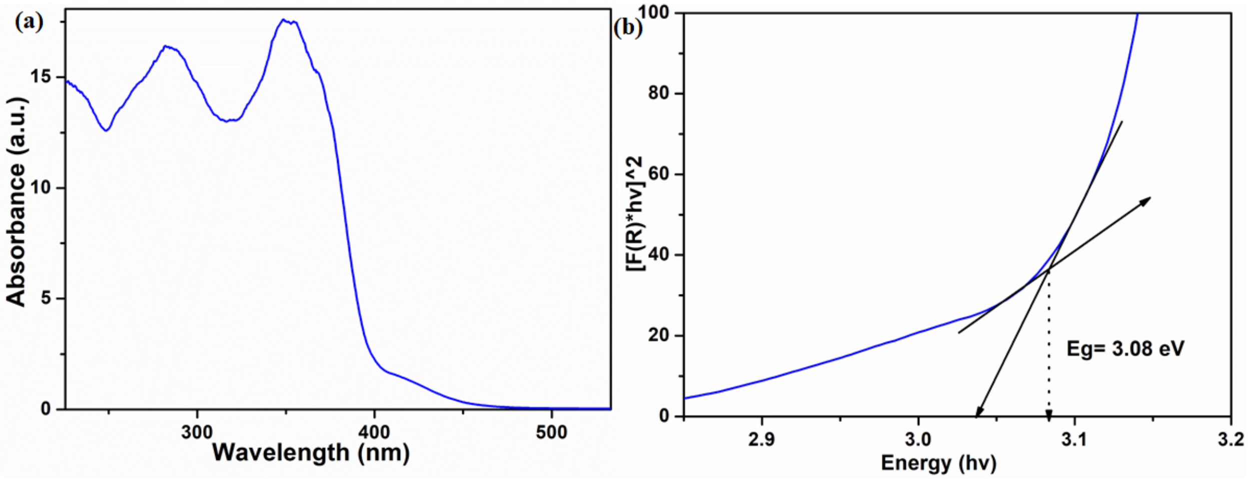

Figure S8. Solid state (a) UV-vis spectrum and (b) band gap energy calculations of H_5L from Kubelka-Munk plot.

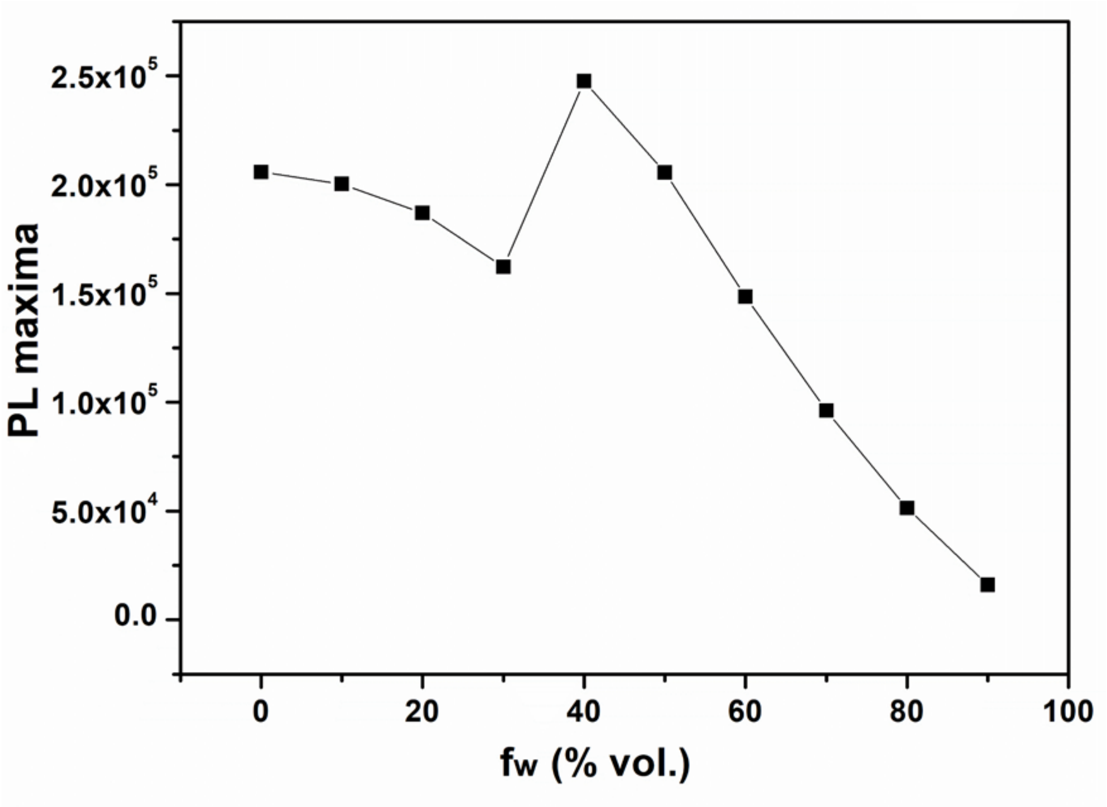

Figure S9. Fluorescence emission intensity plot of H_5L in DMF/H_2O binary mixture.

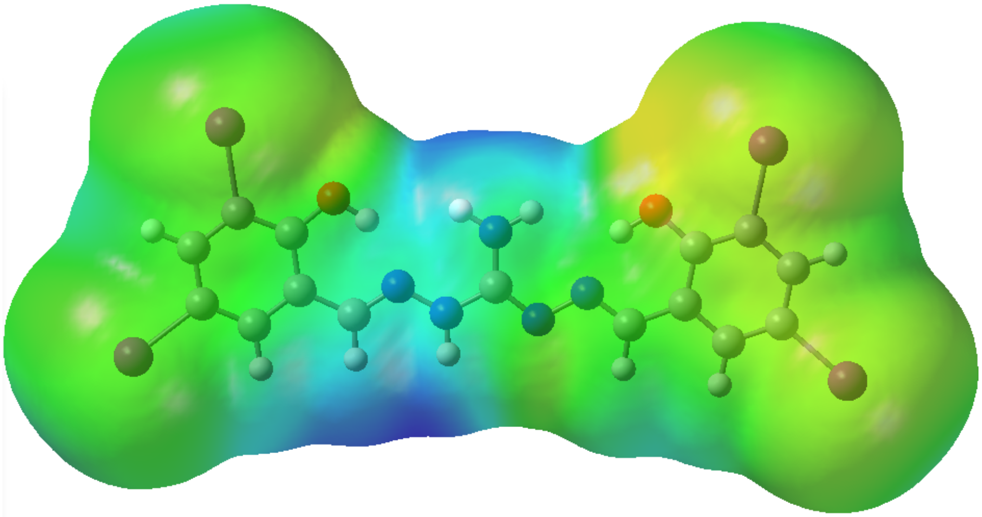

Figure S10. Molecular electrostatic potential maps of the compound.

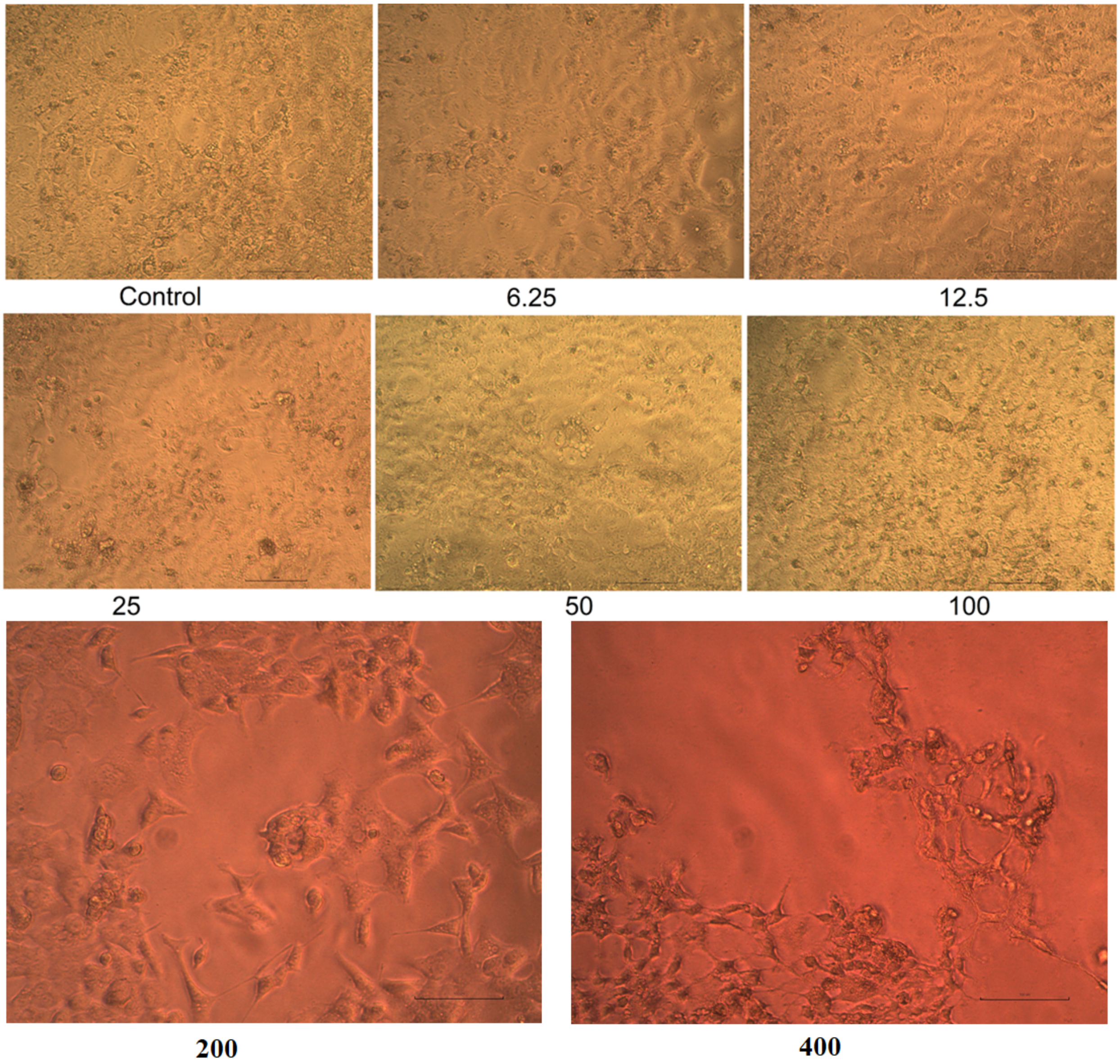

Figure S11. Phase-contrast images of the compound on MCF-7 at different concentrations in μg/mL.

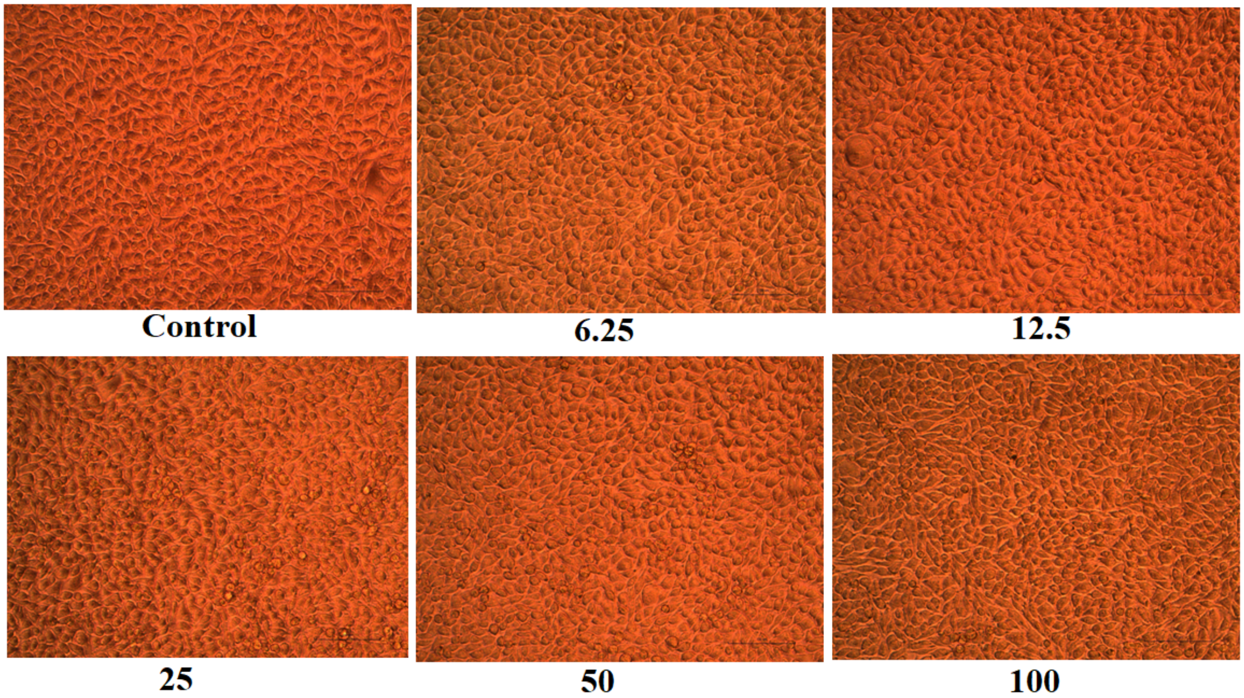

Figure S12. Phase-contrast images of the compound on L929 at different concentrations in µg/mL.

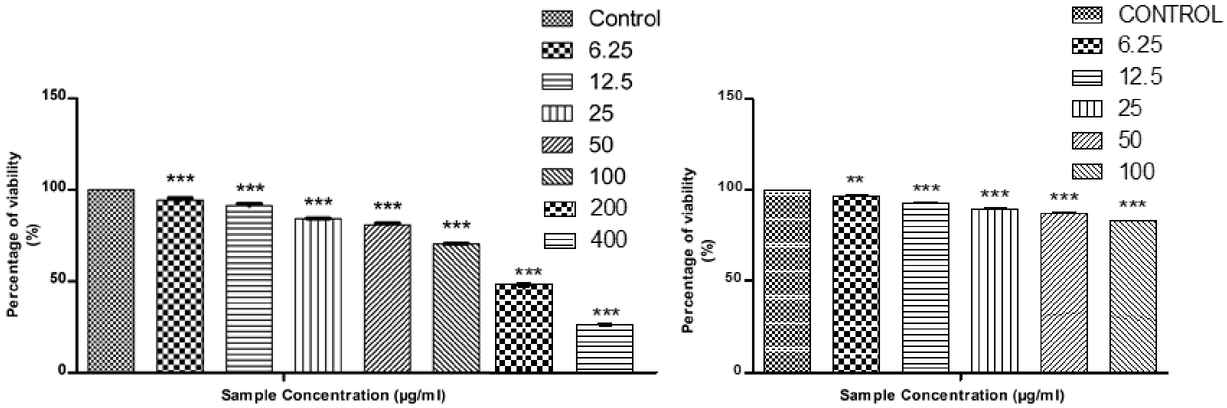

Figure S13: Cytotoxicity effect of H_5L on MCF-7 (left) and L929 cell line (right).

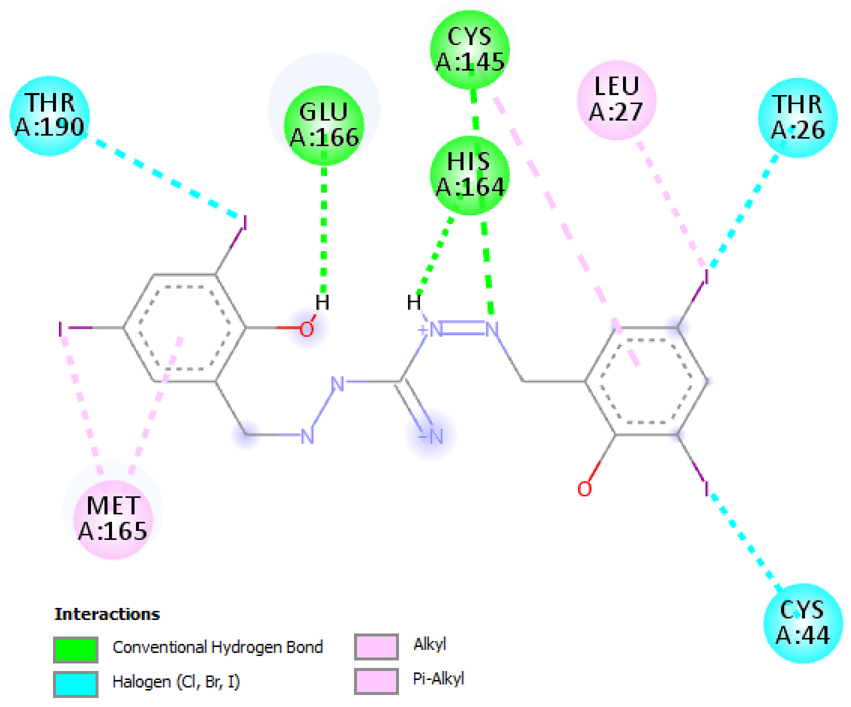

Figure S14. 2D representation of the compound with 6Y2F.

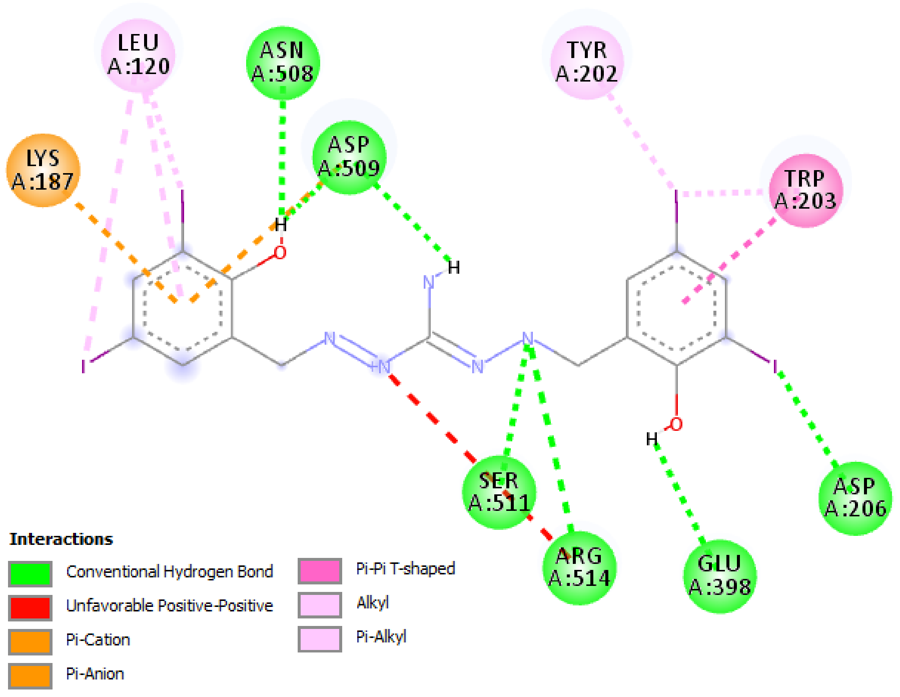

Figure S15. 2D representation of the compound with 6M0J.

Table S1. Selected bond lengths (Å) and bond angles (˚) of H_5L.

SCXRD				DFT			
Bond lengths (Å)		Bond angles (˚)		Bond lengths (Å)		Bond angles (˚)	
C7-N1	1.291(6)	C7–N1–N2	118.5(4)	C7–N1	1.291	C7–N1–N2	118.54
C8-N2	1.362(6)	C3–C4–I2	119.4(3)	C8–N2	1.380	C3–C4–I2	119.36
C8–N3	1.294(6)	O1–C5–C4	118.8(4)	C8–N3	1.305	O1–C5–C4	119.61
C8–N5	1.331(7)	N1–C7–C6	120.6(4)	C8–N5	1.348	N1–C7–C6	123.12
C9–N4	1.287(5)	C9–N4–N3	113.3(4)	C9–N4	1.292	C9–N4–N3	114.78
C5–O1	1.345(5)	N3–C8–N5	126.9(4)	C5–O1	1.341	N3–C8–N5	128.13
C15–O2	1.361(5)	C8–N2–H2A	118(4)	C15–O2	1.337	C8–N2–H2A	115.23
C2–I1	2.094(4)	H5B–N5–H5A	120	C2–I1	2.135	H5B–N5–H5A	119.91
N1–N2	1.352(5)	C3–C2–I1	119.0(3)	N1–N2	1.347	C3–C2–I1	119.30
N3–N4	1.394(5)	C11–C12–I4	120.2(3)	N3–N4	1.373	C11–C12–I4	120.03

Table S2. Interaction parameters of the compound H_5L.

Hydrogen bonding interactions				
D–H ··· A	D–H (Å)	H ··· A (Å)	D–A (Å)	D–H ··· A (°)
C16–H16C ··· O3[a]	0.96	2.56	3.409(6)	147.1
O1–H1A ··· N1	0.82	1.88	2.607(5)	146.7
O2–H2 ··· N4	0.82	1.89	2.614(5)	147.3
N2–H2A ··· O3	0.85(7)	1.94(7)	2.793(6)	174(6)
D = donor, A = acceptor, Equivalent position codes a=-x,-y+1,-z+1				

π ··· π interactions			
Cg ··· Cg	Cg ··· Cg (Å)	α (°)	β (°)
Cg(1) ··· Cg(2)[b]	3.5684(6)	0.000(1)	13.95
Cg(1) ··· Cg(2)[c]	3.5684(6)	0.000(1)	13.95
Cg(1) ··· Cg(2)[d]	3.5684(6)	0.000(1)	13.95
Cg(1) ··· Cg(2)[e]	3.5684(6)	0.000(1)	13.95
Cg(2) ··· Cg(1)[b]	3.5684(6)	0.000(1)	13.95
Cg(2) ··· Cg(1)[c]	3.5684(6)	0.000(1)	13.95
Cg(2) ··· Cg(1)[d]	3.5684(6)	0.000(1)	13.95
Cg(2) ··· Cg(1)[e]	3.5684(6)	0.000(1)	13.95
Equivalent position codes: b= 1-X,-1/2+Y,1-Z, c= 1-X, 1/2+Y,1-Z, d= 1-X, -Y,1-Z, e= 1-X, 1-Y,1-Z, Cg(1)= C(1), C(2), C(3), C(4), C(5), C(6), Cg(2)= C(10), C(11), C(12), C(13), C(14), C(15).			

Iodine bonding interactions	
Y ··· X	Y ··· X (Å)
I1 ··· I2	3.9496(6)
I1 ··· I4	3.8049(6)
I1 ··· I3	4.1596(3)
I1 ··· C13	3.7418(18)

Table S3. The calculated chemical reactivity parameters for the compound (The energy parameters were calculated by assuming -E_{HOMO} as ionization energy and -E_{LUMO} as electron affinity).

Energy parameters (eV)	H_5L
HOMO	-5.913
HOMO-1	-6.328
LUMO	-2.488
LUMO+1	-1.767
E_{HOMO}–E_{LUMO}: ΔE	3.425
Total Energy	-28521
Ionization Energy, I	5.913
Electron Affinity, A	2.488
Chemical hardness, η	1.712
Electronegativity, χ	4.200
Chemical potential, μ	-4.200
Electrophilicity, ω	5.151
Global softness, σ (eV^{-1})	0.291
Nucleophilicity, ε (eV^{-1})	0.194
Dipole moment (Debye)	4.066
Polarizability, α (a.u.)	394.194

Table S4. Percentage viability of MCF-7 and L929 cells with concentration

H_5L Concentration (μg/mL)	% Viability of MCF-7 cell	% Viability of L929 cell
6.25	94.48	96.87
12.5	91.27	92.65
25	84.17	89.62
50	80.91	86.93
100	70.44	83.35
200	48.19	-
400	26.43	-
IC_{50}	181.05	356.54

of the proligand.
